# Ontogenetic transitions, biomechanical trade-offs and macroevolution of scyphozoan medusae swimming patterns

**DOI:** 10.1038/s41598-023-34927-w

**Published:** 2023-06-16

**Authors:** Guilherme M. von Montfort, John H. Costello, Sean P. Colin, André C. Morandini, Alvaro E. Migotto, Maximiliano M. Maronna, Marcelo Reginato, Hiroshi Miyake, Renato M. Nagata

**Affiliations:** 1 Instituto de Oceanografia, Universidade Federal do Rio Grande, Av. Itália, km 8, Rio Grande, RS 96203-000 Brazil; 2 Whitman Center, Marine Biological Laboratory, Biology Department, Providence College, Woods Hole, MA 02543 USA; 3 Marine Biology and Environmental Science, Roger Williams University, Bristol, RI 02809 USA; 4 Departamento de Zoologia, Instituto de Biociências, Universidade de São Paulo, Rua do Matão, Trav. 14, São Paulo, SP 101, 05508-090 Brazil; 5 Centro de Biologia Marinha, Universidade de São Paulo, Rodovia Manuel Hipólito do Rego, km 131.5, São Sebastião, SP 11612-109 Brazil; 6 Departamento de Ciências Biológicas, Universidade Estadual Paulista, Av. Eng. Luiz Edmundo Carrijo Coube, 14-01-Vargem Limpa-Bauru, São Paulo, Brazil; 7 Departamento de Botânica, Instituto de Biociências, Universidade Federal do Rio Grande do Sul, Rio Grande, Brazil; 8 School of Marine Biosciences, Kitasato University, 1-15-1 Kitazato, Minami-ku, Sagamihara, 252-0373 Japan; 9 Biology Department, Providence College, Providence, RI 02918, USA

**Keywords:** Marine biology, Evolutionary developmental biology, Bioenergetics

## Abstract

Ephyrae, the early stages of scyphozoan jellyfish, possess a conserved morphology among species. However, ontogenetic transitions lead to morphologically different shapes among scyphozoan lineages, with important consequences for swimming biomechanics, bioenergetics and ecology. We used high-speed imaging to analyse biomechanical and kinematic variables of swimming in 17 species of Scyphozoa (1 Coronatae, 8 “Semaeostomeae” and 8 Rhizostomeae) at different developmental stages. Swimming kinematics of early ephyrae were similar, in general, but differences related to major lineages emerged through development. Rhizostomeae medusae have more prolate bells, shorter pulse cycles and higher swimming performances. Medusae of “Semaeostomeae”, in turn, have more variable bell shapes and most species had lower swimming performances. Despite these differences, both groups travelled the same distance per pulse suggesting that each pulse is hydrodynamically similar. Therefore, higher swimming velocities are achieved in species with higher pulsation frequencies. Our results suggest that medusae of Rhizostomeae and “Semaeostomeae” have evolved bell kinematics with different optimized traits, rhizostomes optimize rapid fluid processing, through faster pulsations, while “semaeostomes” optimize swimming efficiency, through longer interpulse intervals that enhance mechanisms of passive energy recapture.

## Introduction

Cnidarians are one of the first metazoans to develop locomotion propelled by muscle contractions and their swimming modes are highly associated to the medusae bell shapes^1^. Medusae with more prolate bells, or streamlined bodies, quantified as bell fineness ratio *f* (*f* = H/D, where H = bell height and D = bell diameter), swim via *jet-propulsion* which relies on a robust volume of water to be expelled from inside the bell. On the other hand, medusae with more oblate bells, or flattened bodies, swim via *rowing-propulsion* with the creation of counter-rotating vortices in the wake. The single-layer epitheliomuscular cells that builds the musculature of all cnidarians, however, impose an important physiological constraint to swimming modes of larger sized medusae. Since jet propulsion depends on the volume expelled from the bell cavity, the amount of force produced by these simpler muscular tissues do not scale favourably with size, because the muscles in larger medusae cannot supply sufficient forces required for jet propulsion. Thus, larger medusae species (> 5 cm), in general, have oblate body planes (*f* < 1) and swim using rowing propulsion. Furthermore, oblate bodies tend to produce higher hydrodynamic drag, when compared to the more streamlined ones. However, this shape provides high fluid fluxes, enhancing prey encounter rates through production of feeding currents during pulsations^2–4^. Therefore, bell pulsations both propel the animal forward and generate feeding currents in many scyphomedusae, in a manner that integrates locomotion and feeding functions^1,3^.

Medusae (or jellyfishes) have a saucer- or bell-shaped body called umbrella, in which the underside, known as the subumbrellar cavity, is filled with environmental fluid. This fluid moves alternately in and out of the medusa bell during pulsation, creating a series of interconnected vortices, owing to fine control of its flexible bell margin^1,3^. Swimming thrust involves the contraction of the single-layer epithelial circular muscle that reduces the subumbrellar volume and forces the fluid out of the subumbrellar space^3,5,6^. During this phase, a “starting vortex” is formed around the bell margin and channels fluid downwards^3^. Subsequently, subumbrellar muscles relax, and due to antagonistic interactions of elastic fibres of the mesoglea that store energy of bell contraction, the bell returns to its expanded, or relaxed shape, as fluid refills the subumbrellar cavity^7^. At this phase, a “stopping vortex” is formed inside the bell, with opposite rotation in relation to the starting vortex. Stopping vortices positioned in the subumbrellar cavity increases pressure and pushes the bell forward. This passive motion, due to the elastic recoil, is followed by short pauses in bell motion between subsequent pulsations, in which a secondary velocity peak is observed^8^. The passive locomotion during such pauses, called passive energy recapture (PER), can contribute for more than a third of total travelled distances during the pulsation cycle, in species of the genus *Aurelia* Lamarck, 1816^6,8,9^. Contraction of the bell is usually faster than relaxation, and this asymmetry between phases results in greater fluid velocities, consequent momentum transfer to the fluid that generates medusa movement. Thus, medusae swimming operates in a pulsed, unsteady motion between contraction and relaxation of the bell^5^.

Scyphomedusae (class Scyphozoa) are exclusively marine organisms divided into two monophyletic lineages: Coronatae Vanhöffen, 1892 (= Coronamedusae, 59 spp.) and Discomedusae (172 spp.)^10^. Discomedusae is composed of "Semaeostomeae" L. Agassiz, 1862 (84 spp.) and Rhizostomeae Cuvier, 1800 (88 spp.)^11^. Meanwhile, "Semaeostomeae" is considered paraphyletic with respect to Rhizostomeae^12,13^. The initial medusa stage of Scyphozoa, the ephyra, is morphologically similar among the different lineages within the class^14,15^. Ephyrae are only a few millimetres in size, whereas juveniles and adults are usually larger in diameter (> 2 cm), reaching over a meter in species of the genera *Cyanea* Péron & Lesueur, 1810 and *Nemopilema* Kishinouye, 1922. During early development (4 to ~ 20 mm), the ephyra lacks specialized feeding structures (tentacles and oral arms) and has a clefted umbrella margin, with 8 marginal lobes^15^. Ephyrae pulse more frequently (> 3 Hz) than adult medusae^4,16–18^. Due to their size, ephyrae live in an environment dominated by viscous forces (Reynolds Number, Re < 10), where pulsations are rapidly decelerated and fluid transport against capture structures is restricted, without production of circulating vortices in their wakes^4,17,18^. As they grow, the umbrella margin becomes continuous and food capture structures (tentacles and oral arms) are formed. Inertial forces (Re > 100) become increasingly dominant, and the medusae begin to generate more complex flows through pulsation. Although in species of *Aurelia* the ephyrae tend to have a more prolate shape than their juveniles and adults^19^, for most species the adult medusae have a more prolate body plan (higher fineness ratio) than their ephyrae^4^. Since body shapes are related to drag and added mass effect, that can reduce swimming efficiency. It is expected that ontogenetic transitions in scyphomedusae should have implications for swimming performance, foraging modes, and mechanisms used for encountering and capturing evasive prey^4,16,20^. These factors may be determinants for trophic diversification of scyphomedusae^1^.

Although bell pulsations are important for foraging among Discomedusae (Rhizostomeae and “Semaeostomeae” sub-clades), little is known about how differences in bell shape and relative timing of each pulsation phase are reflected in performance and swimming patterns. Since those parameters are quite variable among the few species of larger medusae already studied^4,8,9,19,21^, this raises questions about phylogenetic signals in swimming kinematic patterns, and other functional implications. Besides the low phylogenetic representativeness of these studies [*Chrysaora quinquecirrha* (Desor, 1848)*, Cyanea capillata* (Linnaeus, 1758)*, Aurelia aurita* (Linnaeus, 1758), *Stomolophus meleagris* Agassiz, 1860, *Lychnorhiza lucerna*, *Catostylus mosaicus* (Quoy & Gaimard, 1824), *Phyllorhiza punctata* von Lendenfeld, 1884)] there are also the small sample sizes and size range of individuals, mainly due to the inaccessibility of healthy animals for experimental assays. Still with these limitations, it is possible to recognise some swimming patterns. Medusae of “Semaeostomeae” (n = 3 spp. studied) tend to pulse at lower frequencies (< 1 Hz), with slower contraction and relaxation cycles^2,17,18,22,23^. By contrast, medusae of the order Rhizostomeae (n = 4 spp. studied) appear to have more robust swimming patterns, pulsing at higher frequencies (~ 2 Hz), and reaching higher velocities and Reynolds numbers than other scyphomedusae of similar size diameter^4,16,23^. These apparent differences may have important functional and ecological implications for prey selectivity, because the strength of the feeding currents affects the type of entrained prey^4,22^. Likewise, feeding performances may be affected, because the volume of cleared water is associated with the amount of entrained water through bell pulsations^24–26^. Bioenergetics may also be affected because mechanisms such as passive energy recapture (described above) and virtual wall effect, that represents the interaction between the stopping and starting vortex, creating an effect similar to a near solid boundary, influence swimming efficiency and reduce the energetic cost of transport^6,8,9,21^.

Evolutionary processes have selected for highly energetic efficient swimming modes, with some scyphomedusae having the lowest metabolic cost of transport (J kg^−1^ m^−1^) among all metazoans^8^. Swimming kinematics and performances have long been explored in scyphomedusae^5,8,21,27,28^. However, only recently, detailed fluid dynamics processes involving medusae propulsion have been elucidated (i.e., passive energy recapture, suction thrust, and virtual wall effect) due to advances in flow visualization and modelling techniques, in association with empirical observations^6^. Nevertheless, taxonomic sampling of swimming modes is still limited, hampering broader comparisons of swimming patterns between different scyphomedusae phylogenetic lineages (e.g., the main clades Coronamedusae and Discomedusae). This also prevents the elucidation of possible relationships between such swimming patterns, the group wide morphological variance, as well as about functional consequences of morphological development.

In this context, high-speed imaging is a powerful tool for understanding the functioning of the locomotion and food-gathering mechanisms of medusae^2–4,6,16,18,19,22,29,30^. Given the important ecological roles of scyphomedusae in the marine realm, a comparative investigation, applying phylogenetics, biomechanical and kinematic analyses could reveal developmental patterns underlying scyphozoan lineages, aiding in understanding about functional morphology and kinematic variability that underlie swimming behaviour, energy costs and foraging modes.

Here we describe biomechanical and kinematic parameters of swimming within distinct lineages of scyphomedusae, at different developmental stages, using high-speed images. Specifically, we aimed: (1) to describe effects of ontogenetic transition on bell shape, bell kinematics and swimming performance in scyphozoan lineages; (2) to describe possible patterns in the kinematics and biomechanics of swimming in scyphozoan lineages; and (3) to evaluate possible phylogenetic signals on swimming patterns underlying scyphozoan lineages. We propose two hypotheses: first, through ontogeny there is a transition in bell kinematics and swimming performances that can be predicted by bell diameter and differs between the orders “Semaeostomeae” and Rhizostomeae. Second, when parameters of bell kinematics and swimming performances are normalized by size, these will reveal phylogenetic signals and conserved swimming patterns among closely related species. Hence, we discuss how swimming parameters are associated to other functional features as energetic costs of transport and feeding mechanics.

## Results

### Jellyfish swimming dataset

A total of 109 image sequences of 17 species (11 families and 13 genera, see Supplementary Fig. S1) of Scyphozoa were used, from both recorded sequences (n = 81) and compiled data from literature (n = 28). Analysed species represent, as much as possible, the main scyphozoan clades: only a single sequence of one Coronamedusae [family Linuchidae: *Linuche unguiculata* (Swartz, 1788)] was included and 108 sequences of 16 Discomedusae species [50 sequences of eight “Semaeostomeae” species representing three families: Pelagiidae—*Sanderia malayensis* (Goette, 1886), *Chrysaora pacifica* (Goette, 1886), *C. lactea*, *C. quinquecirrha,* and *C. plocamia* (Lesson, 1830), Cyaneidae—*C. capillata*, and Ulmaridae—*Aurelia solida* Browne, 1905, *A. coerulea* von Lendenfeld, 1884; and 58 sequences of eight Rhizostomeae species representing seven families: Stomolophidae—*S. meleagris*, Rhizostomatidae—*Rhopilema esculentum* Kishinouye, 1891, Lychnorhizidae*—L. lucerna*, Catostylidae—*C. mosaicus*, Cepheidae—*Cotylorhiza tuberculata* (Macri, 1778), Leptobrachidae—*Thysanostoma thysanura* Haeckel, 1880 and Mastigiidae—*P. punctata* and *Mastigias papua* (Lesson, 1830)]. Our dataset, with the number of specimens recorded (either from new data or from the literature), their bell diameter ranges, and the origin of the medusae culture are described in the Table 1. We recorded for the first-time high-speed sequences for the rhizostome species *T. thysanura*, *R. esculentum*, *C. tuberculata*, and the “semaeostome” species *C. lactea*, *C. pacifica* and *C. plocamia*.

**Table 1:** Analysed scyphozoan species, with number of recorded and extracted (n) sequences, obtained from the literature (overwritten), the range of minimum and maximum bell diameter (D_min_ – D_max_).

Order	Family	Species	n (recorded + extracted^source^)	D_min_ − D_max_ (cm)	Origin of the culture
Coronatae	Linuchidae	*Linuche unguiculata* (Swartz, 1788)	1^2^	1.5	Monterey Bay Aquarium (USA)
“Semaeostomeae”	Cyaneidae	*Cyanea capillata* (Linnaeus, 1758)	9 (7 + 2^b,f^)	0.51–3.9	Monterey Bay Aquarium (USA)
	Pelagiidae	*Chrysaora quinquecirrha* (Desor, 1848)	11 (10 + 1^c^)	0.22–5.45	Woods Whole (Marine Biological Laboratory, USA)
		*Chrysaora plocamia* (Lesson, 1830)	4	0.5–0.79	Ilo (Peru)
		*Chrysaora pacifica* (Goette, 1886)	4	0.7–2.67	
		*Chrysaora lactea* Eschscholtz, 1829	1	6.05	Ubatuba (Brazil)
		*Sanderia malayensis* (Goette, 1886)	5	1.27–2.09	Monterey Bay Aquarium (USA)
	Ulmaridae	*Aurelia solida* Browne, 1905	4	0.3–0.47	Philippines
		*Aurelia coerulea* von Lendenfeld, 1884	12 (9 + 3^a,b,e^)	1.46–4.78	Monterey Bay Aquarium (USA)
Rhizostomeae	Cepheidae	*Cotylorhiza tuberculata* (Macri, 1778)	8	0.19–4.22	Spain
	Mastigiidae	*Phyllorhiza punctata* von Lendenfeld, 1884	5 (4 + 1^d^)	0.41–2.8	Dr. Gerhard Jarms, Berlin Zoo (Germany)
		*Mastigias papua* (Lesson, 1830)	9	0.34–3.44	Dr. Gerhard Jarms, Berlin Zoo (Germany)
	Leptobrachidae	*Thysanostoma thysanura* Haeckel, 1880	2	0.29	Enoshima (Japan)
	Lychnorhizidae	*Lychnorhiza lucerna* Haeckel, 1880	19^g^	0.63–6.14	Cananéia (Brazil)
	Catostylidae	*Catostylus mosaicus* (Quoy & Gaimard, 1824)	8	0.37–9.42	Australia
	Stomolophidae	*Stomolophus meleagris* Agassiz, 1860	3 (2 + 1^b^)	1.61–3.75	Gulf of Mexico (USA)
	Rhizostomatidae	*Rhopilema esculentum* Kishinouye, 1891	4	0.73–5.54	Japan

The studies from which kinematic data were obtained were: ^a^Costello and Colin (1994); ^b^Costello and Colin (1995); ^c^Ford et al. (1997); ^d^D'ambra et al. (2001); ^e^MacHenry and Jed (2003); ^f^Higgins et al. (2008); and ^g^Nagata et al. (2016).

We acknowledged that the “Semaeostomeae” group was not recovered as monophyletic in most of recent phylogenetic analysis and in our own analysis^12,31^; because there is not a clear hypothesis to replace nor update it, we choose to keep it for the sake of simplicity.

### Ontogenetic transitions of biomechanical and swimming kinematics variables

**Figure 1:**
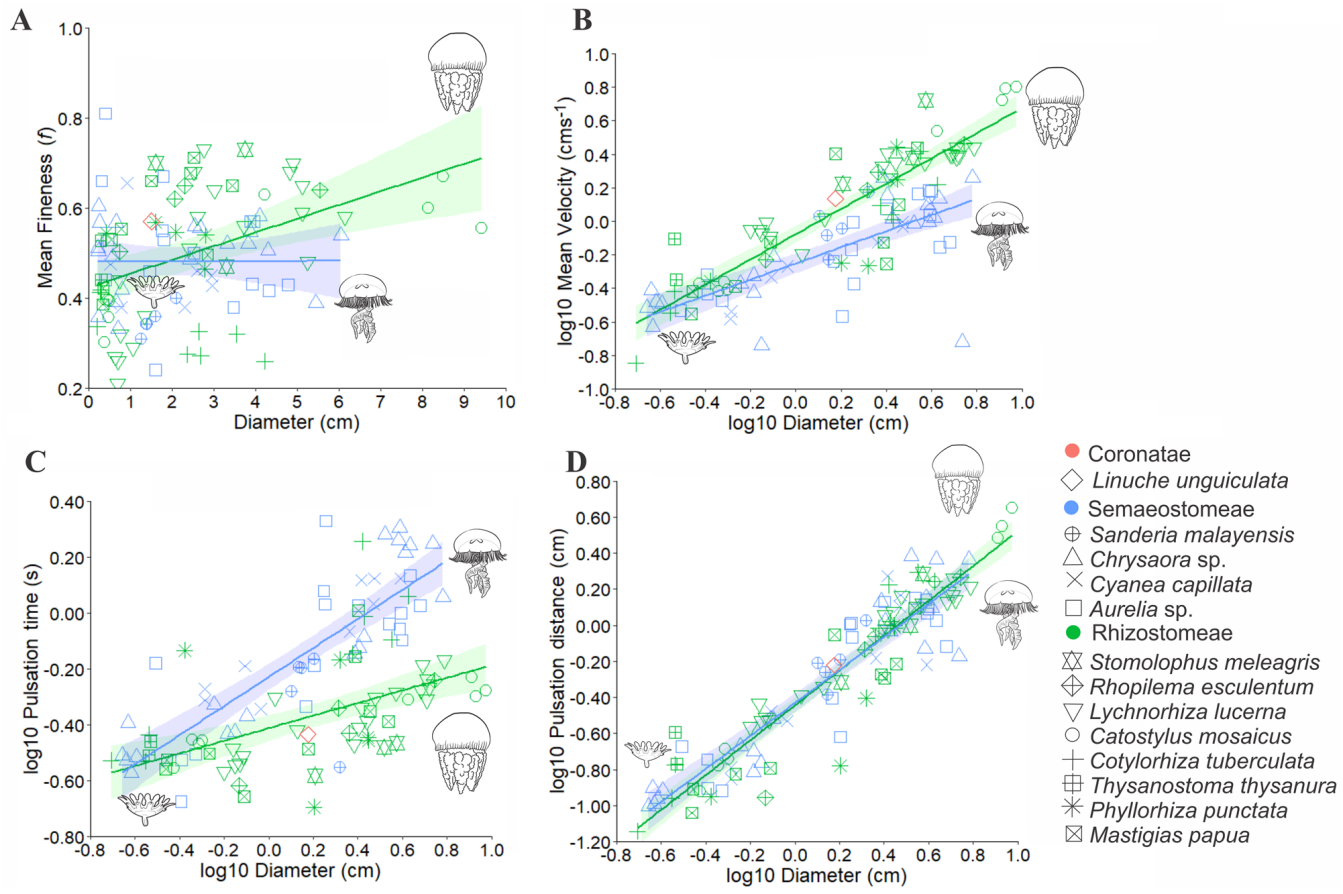
Scyphozoa orders relationship between diameter (cm) and: (A) mean fineness; (**B**) mean velocities; (**C**) pulsation time; and (**D**) pulsation distance; label displays the shape of each plotted species. In red is the Coronatae = *Linuche*; in blue are the “Semaeostomeae” = *Sanderia*, *Chrysaora*, *Cyanea* and *Aurelia*; in green are the Rhizostomeae = *Stomolophus*, *Rhopilema*, *Lychnorhiza*, *Catostylus*, *Cotylorhiza*, *Thysanostoma*, *Phyllorhiza,* and *Mastigias*. Images of representative morphologies were used, common generalized ephyrae, *Aurelia* for “Semaeostomeae” and *Catostylus* for Rhizostomeae.

Scyphomedusae had a typically flattened or oblate bell with *f*_*mean*_ < 1 (Fig. 1A). In medusae of “Semaeostomeae”, no significant effect of body size on *f*_*mean*_ was found. This indicates that each species has a particular pattern of bell shape development that cannot be predicted by pooling species of this order together. In medusae of Rhizostomeae a weak positive correlation between *f*_*mean*_ and body size was found as the more flattened ephyrae (*f*_*mean*_ < 0.4) continuously developed a more streamlined bell (*f*_*mean*_ > 0.6) in juveniles (> 6 cm).Scyphozoan mean (*u*_*mean*_) and maximum swimming velocities (*u*_*max*_) (Fig. 1B, Supplementary Fig. S2A) increase with size for both orders. From early ephyrae (< 1 cm), Rhizostomeae medusae swam slightly faster (*u*_*mean*_ = 0.8 cm s^−1^), than those of “Semaeostomeae” (*u*_*mean*_ = 0.5 cm s^−1^). Velocity differences between these groups became clearer in greater diameters, as 6 cm “Semaeostomeae” medusae swim at *u*_*mean*_ = 1.5 cm s^−1^, whereas Rhizostomeae medusae of the same size swim at *u*_*mean*_ = 3.5 cm s^−1^. The only Coronatae medusa (1.5 cm, *L. unguiculata*) swims at velocities comparable with those of Rhizostomeae species (*u*_*mean*_ > 1.2 cm s^−1^). Relatively faster velocities of *C. pacifica* and *C. lactea* (*u*_*mean*_ > 1.8 cm s^−1^) stood out among the “Semaeostomeae” medusae, whereas those of the rhizostomes *T. thysanura* ephyrae (*u*_*mean*_ = 0.75 cm s^−1^) and *S. meleagris* (4 cm, *u*_*mean*_ > 5 cm s^−1^) were higher than all other scyphozoan medusae of the same size (Supplementary Fig. S7).

Reynolds number (mean *Re*_*mean*_ and max *Re*_*max*_) followed the pattern of swimming velocities, increasing predictably with body size in both orders (Supplementary Fig. S2B,C). In both orders, early ephyrae (< 1 cm) swam in a viscous dominated environment (*Re*_*mean*_ < 100), and from 0.6 cm on, Rhizostomeae ephyrae had higher *Re* (*Re*_*mean*_ = 25) than those of the “Semaeostomeae” (*Re*_*mean*_ = 15). These differences became clearer, as 6 cm “Semaeostomeae” medusae swam at *Re*_*mean*_ ~ 500, whereas the same sized Rhizostomeae medusae reached *Re*_*mean*_ ~ 1500.

Pulsation frequencies (*P*_*freq*_) and times (*P*_*time*_) were negatively and positively correlated with bell diameter (Supplementary Fig. S2D, Fig. 1C). Recently released ephyrae (~ 0.3 cm) of both orders pulse at similar rates (*P*_*freq*_ ~ 3.8 Hz and *P*_*time*_ ~ 0.26 s). However, from 0.6 cm, a clear distinction was observed, with “Semaeostomeae” medusae exhibiting lower pulsation frequencies (*P*_*freq*_ ~ 2.2 Hz) and higher pulsation times (*P*_*time*_ ~ 0.41 s), compared to Rhizostomeae medusae (*P*_*freq*_ ~ 2.88 Hz and *P*_*time*_ ~ 0.31 s). This pattern became clearer through development, as 6 cm “Semaeostomeae” medusae pulse at *P*_*freq*_ ~ 0.6 Hz (*P*_*time*_ ~ 1.5 s), while Rhizostomeae pulse at *P*_*freq*_ ~ 1.5 Hz (*P*_*time*_ ~ 0.6 s). Some exceptions were found in the rhizostome *C. tuberculata*, that had an ontogenetic pulsation pattern that resembled those of “Semaeostomeae” species, particularly those of the Pelagiidae family (Supplementary Figs. S8, S9).

Pulsation distance (*P*_*dist*_) increased with diameter in both orders (Fig. 1D). However, unlike other parameters, a high overlap of values between species of both orders indicated similar mean distances reached per pulsation. All “Semaeostomeae” species had steadier values in medusae > 4 cm, with P_*dist*_ ~ 1 cm for species of Ulmaridae and Cyaneidae and *P*_*dist*_ ~ 1.5 cm for species of Pelagiidae. By contrast, Rhizostomeae species continue to increase pulsation distances (*P*_*dist*_ > 2 cm) in greater sizes (> 6 cm) (Supplementary Fig. S11).

Contraction times (C_*time*_) and distance travelled during contraction (*C*_*dist*_) increased with bell diameter in both orders (Supplementary Fig. S2E,F). From early development up to 2 cm, species of both orders followed similar patterns. Then, in larger “Semaeostomeae” medusae (> 6 cm) values observed were *C*_*time*_ ~ 0.4 s and *C*_*dist*_ ~ 0.5 cm, whereas for Rhizostomeae medusae of the same size, *C*_*time*_ ~ 0.2 s and *C*_*dist*_ > 0.8 cm. The rhizostome *C. tuberculata* differed from the pattern found within its order, because of higher contraction times (*C*_*time*_ > 0.25 s in 4 cm bell diameter) resembling those found in “Semaeostomeae” (Supplementary Fig. S12). The rhizostome *C. mosaicus* together with *S. meleagris* reached the highest contraction distances among all scyphozoan species (> 4 cm, *C*_*dist*_ > 0.8 cm) (Supplementary Fig. S13).

Relaxation times (*R*_*time*_) and distances travelled during relaxation (*R*_*dist*_) increased with bell diameter in both orders (Supplementary Fig. S3A,B). Recently released ephyrae (0.3 cm) had similar relaxation patterns (*R*_*time*_ ~ 0.12 s and *R*_*dist*_ ~ 0.05 cm). Then, especially for *R*_*time*_, from 0.6 cm on, “Semaeostomeae” species spent more time in the relaxation (*R*_*time*_ ~ 0.25 s, and *R*_*dist*_ ~ 0.31 cm) than species of Rhizostomeae (*R*_*time*_ ~ 0.15 s and *R*_*dist*_ ~ 0.25 cm.) In larger diameters, these differences were enhanced.

Interpulse time (*I*_*time*_) was not explained by medusae diameter for both orders (p > 0.05) (Supplementary Fig. S3C), whereas interpulse distance (*I*_*dist*_) and passive energy recapture (PER) showed little correlation with diameter) (Supplementary Fig. S3D,E). Ephyrae (< 2 cm) generally do not present *I*_*dist*_ and PER, because of the high pulsation frequency without pauses between pulsations. Higher interval times and contributions of PER (> 0.8 s and > 0.2) were observed in the “Semaeostomeae” families Ulmaridae, Pelagiidae, and Cyaneidae, and in the rhizostome *C. tuberculata* (Cepheidae) (Supplementary Figs. S15, S17). Some larger individuals of *R. esculentum* (> 5 cm) and *L. lucerna* also had high *I*_*time*_ ~ 0.25 s, *I*_*dist*_ ~ 0.4 cm and 0.3 cm, and greater contributions of PER > 0.2, respectively (Supplementary Figs. S16, S17, S18).

### Relationships between kinematic and biomechanical variables and swimming patterns of Scyphozoa

**Figure 2:**
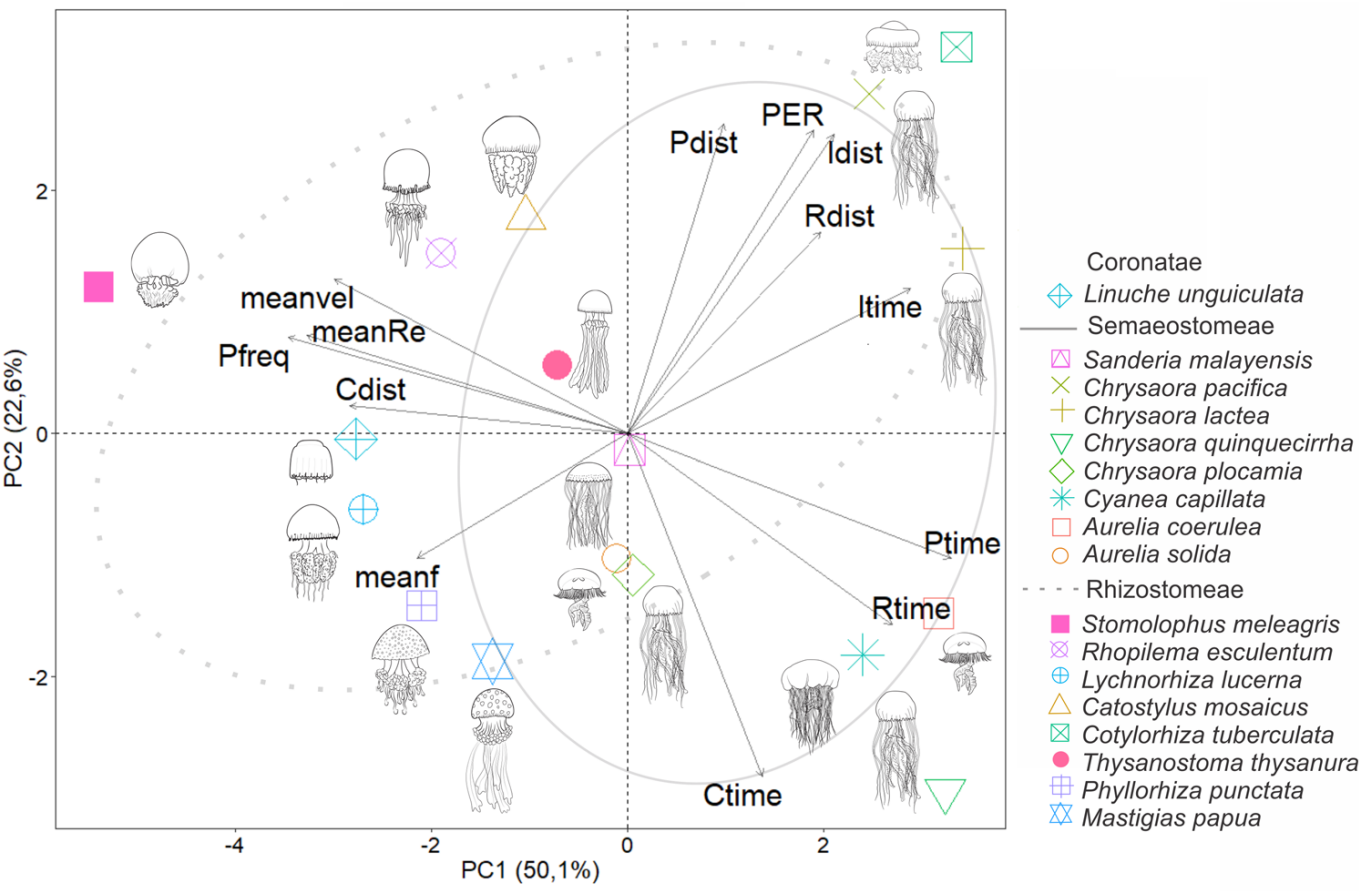
Principal component analysis showing the relationships among Scyphozoa swimming for size-normalized biomechanics and kinematic variables. Ellipses represent confidence intervals of 80%. Label displays the shape of each plotted species. *Meanf* mean fineness (*f*), *meanvel* mean velocity (cm s^−1^), *meanRe* mean Reynolds (*Re*), *Pfreq* pulsation frequency (Hz), *Ptime* pulsation time (s), *Pdist* pulsation distance (cm), *Ctime* contraction time (s), *Cdist* contraction distance (cm), *Rtime* relaxation time (s), *Rdist* relaxation distance (cm), *Itime* interpulse time (s), *Idist* interpulse distance (cm), *PER* passive energy recapture (cm).

Most of the variation in our data was explained by the first two principal components (PCs), which accounted for 72.7% of the variance (PC1 = 50.1% and PC2 = 22.6%) (Fig. 2). The variables contributions and correlations of the first four PCs are shown in Supplementary Table S2.

Species of “Semaeostomeae” were positively correlated with PC1, because of their longer and paused pulsation patterns. By contrast, most of Rhizostomeae species were negatively correlated to PC1 because of their higher swimming performance, except for *C. tuberculata*. The “semaeostome” species *C. pacifica*, *C. lactea*, and the rhizostome *C. tuberculata* were positively associated with PC2, because of their higher pulsation distances, interpulse times, and greater use of passive energy recapture.

**Figure 3:**
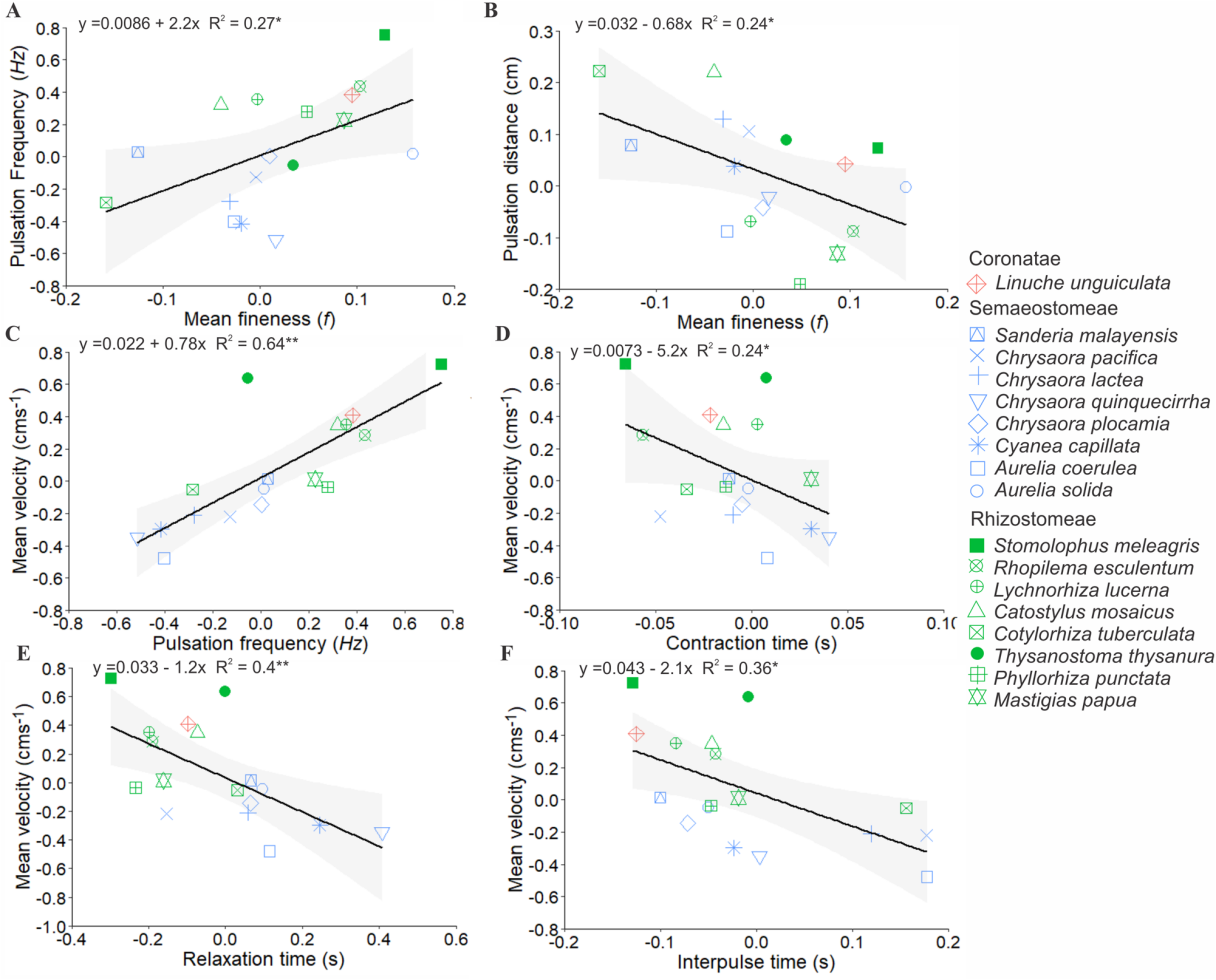
Regressions showing the relationships of size-normalized swimming variables. Related with alterations on the shape of the bell (fineness *f*) (**A**) pulsation frequency and (**B**) pulsation distance; and over velocities related with: (**C**) pulsation frequency; (**D**) contraction time; (**E**) relaxation time; (**F**) interpulse time. Label displays the shape of each plotted species. Coronatae = *Linuche*; “Semaeostomeae” = *Sanderia*, *Chrysaora*, *Cyanea* and *Aurelia*; Rhizostomeae = *Stomolophus*, *Rhopilema*, *Lychnorhiza*, *Catostylus*, *Cotylorhiza*, *Thysanostoma*, *Phyllorhiza* and *Mastigias*.

Relationships among size-normalized swimming variables (Fig. 3) indicated that more oblate bell shapes (i.e. lower *f*_*mean*_) were associated to lower pulsation frequencies (Fig. 3A), and higher pulsation distances (Fig. 3B). Medusae with higher velocities also pulses at higher frequencies (Fig. 3C), specifically due to shortened contraction (Fig. 3D), relaxation (Fig. 3E) and interpulse times (Fig. 3F).

### Phylogenetic signal and ancestral character reconstruction of Scyphozoa swimming

Estimated phylogenetic signal (Table 2) demonstrated that swimming variables such as Reynolds number, pulsation frequency, relaxation time and pulsation time had strong phylogenetic signal, implying that values tend to be similar in related species owing to their shared evolutionary history. Although the last three variables did not presented p < 0.05, this is probably related to the low number of terminals in the tree (n = 17), whereas high K values (K > 0.85) support the strong phylogenetic signal. Interpulse time, relaxation distance and pulsation distance had weak phylogenetic signal, that indicates frequent alterations independently evolved across the phylogeny.

**Table 2:** Size normalized estimates of phylogenetic signal in Scyphozoa swimming variables.

Variable	K	p
Mean Reynolds (*Re*)	1.04	0.020
Pulsation frequency (*Hz*)	0.89	0.073
Relaxation time (s)	0.88	0.089
Pulsation time (s)	0.86	0.082
Contraction distance (cm)	0.82	0.132
Mean velocity (cm s^−1^)	0.80	0.168
Mean fineness (*f*)	0.74	0.306
Passive energy recapture	0.74	0.316
Contraction time (s)	0.73	0.302
Interpulse distance (cm)	0.73	0.295
Interpulse time (s)	0.69	0.338
Relaxation distance (cm)	0.61	0.619
Pulsation distance (cm)	0.57	0.714

The significance value of each is calculated by phylosig for a randomization test (K).

**Figure 4:**
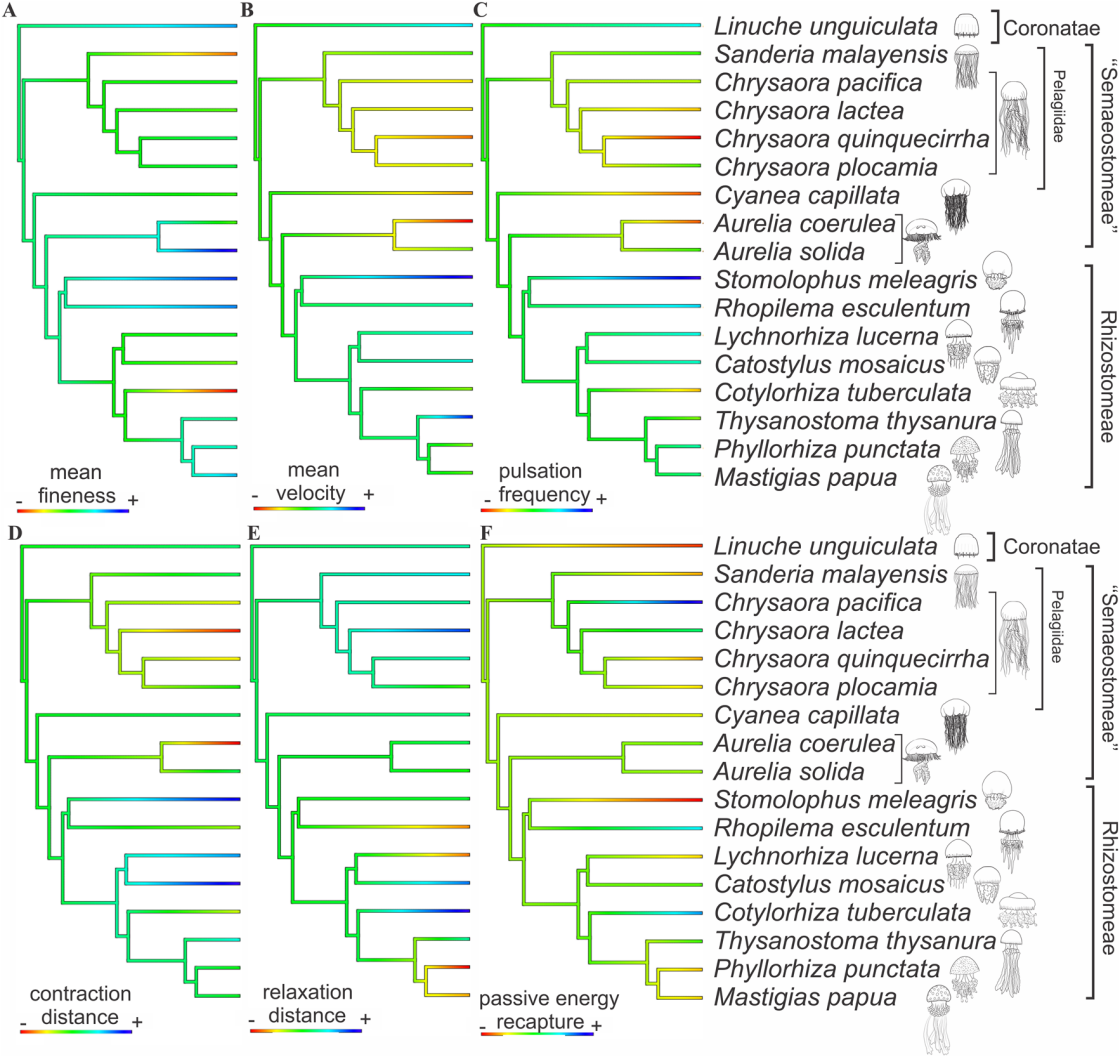
Ancestral character reconstructions estimated from size-normalized bell kinematics and swimming performances of scyphozoan medusae: (**A**) mean fineness; (**B**) mean velocity; (**C**) pulsation frequency; (**D**) contraction distance; (**E**) relaxation distance; (**F**) passive energy recapture (PER). Warm and cold colours on the scale for each variable represent higher and lower values, respectively.

Size-normalized ancestral reconstruction for *f*_*mean*_ (Fig. 4A) did not indicate a clear phylogenetic pattern for the evolution of bell shapes and is suggestive of intermediate *f*_*mean*_ for most ancestral nodes, which was also conserved in most species. Nonetheless, a few shifts were estimated. Highly streamlined shapes (higher *f*_*mean*_) have evolved independently in *L. unguiculata* (Coronatae), *A. solida* (“Semaeostomeae”), and in *S. meleagris* and *R. esculentum* (Rhizostomeae). While the most oblate shapes evolved independently in *S. malayensis* (“Semaeostomeae”) and *C. tuberculata* (Rhizostomeae).

Swimming velocity (*u*_*mean*_), pulsation frequency (*P*_*freq*_), and contraction distances (*C*_*dist*_) (Fig. 4B–D, respectively) further corroborate the correlation of these variables and point to a scenario of different topologies between “Semaeostomeae” with the lowest values, and Rhizostomeae with the highest. The “Semaeostomeae” species *C. lactea*, *A. coerulea, C. pacifica*, and *C. quinquecirrha* had the lowest values among Scyphozoa, while the Rhizostomeae species *S. meleagris*, *C. mosaicus*, and *L. lucerna* the highest.

Ancestral estimations for relaxation distance (*R*_*dist*_) (Fig. 4E) further point to different evolutionary paths, where reached distances during this phase were in general smaller in Rhizostomeae than in “Semaeostomeae”. Passive energy recapture ancestral estimation shows recurrent alterations through the phylogeny (Fig. 4F), with the “semaeostomes” *C. pacifica* and *C. lactea*, and the rhizostome *C. tuberculata* and *R. esculentum* showing the highest PER among Scyphozoa.

## Discussion

We have quantified and compared the swimming patterns of several different species of scyphomedusae and described for the first time the swimming behaviour of *T. thysanura*, *C. lactea*, *C. pacifica*, *C. plocamia*, *R. esculentum*, and *C. tuberculata*. We have shown, through a detailed description of the ontogenetic transitions of bell kinematics and swimming performances in medusae of Scyphozoa that: (1) size-effects on swimming parameters were highly dependent of the orders “Semaeostomeae” and Rhizostomeae, however, the distance travelled per pulse did not differ between the groups; (2) swimming kinematics and performance are conserved for the Rhizostomeae but more variable among “Semaeostomeae” species (which may reflect the well accepted paraphyly within “Semaeostomeae”^12,32–34^). Thus, both hypotheses 1 and 2 were accepted.

Despite the similarities in ephyra body plans, morphological and functional variations emerge through early ontogeny (e.g.^15^) and such transitions also affect swimming and feeding behaviour^4^. Ephyrae swimming is characterized as a drag-based paddling^18^, a common strategy for animals performing in low Reynolds number fluids^35^. As juveniles and adults, medusae use rowing propulsion which relies more on inertial vortex interactions. Rowing uses a fine control of bell kinematic to create counterrotating vortices that generate forward thrust and allows manoeuvrability, while also channelling fluid through feeding structures^3,4,9^. Our findings demonstrate that Rhizostomeae transition to the inertia dominated fluid regime (*Re* > 100) at a smaller size (~ 1.3 cm) than “Semaeostomeae” (2 cm; Supplementary Fig. S2B). Furthermore, corroborating previous isolated observations^2,4,36,37^, larger rhizostome medusae (> 6 cm) had more than twofold higher swimming performances, in terms of velocity (*u*_*mean*_ = 3.5 cm s^−1^) and Reynolds number (*Re*_*mean*_ ~ 1500), than similarly sized “semaeostomes” (*u*_*mean*_ = 1.5 cm s^−1^ and *Re*_*mean*_ ~ 500) (Fig. 1B, Supplementary Fig. S2B).

The observed earlier transition by rhizostome to the inertial fluid regime corresponds to an earlier metamorphose of rhizostome ephyrae than “semaeostomes”^15^. Jordano et al. showed that many rhizostomes filled in the space between their ephyrae lappets to make a continuous bell at a smaller size. Rhizostomes, in the study, also started to develop oral arms at a smaller size than the “semaeostomes”. This is likely associated to the different kinematics that we observed which increased the Re around ephyrae earlier in development and, it has been shown, that metamorphosis by ephyrae to adult bell forms can be triggered by their surround fluid regime^38^. These differences also imply that rhizostome medusae may undergo earlier transitions on diet, since the ability to entrain more evasive prey rely on the strength of vortices produced by bell pulsations (among other variables), that also scale with increasing size and swimming velocities^4,16^.

Higher swimming performance by rhizostomes was achieved by having much more rapid bell kinematics. Rhizostomes had higher pulsation frequencies (1.5 Hz vs. 0.6 Hz), shorter contraction and relaxation times which resulted in much shorter overall pulsation times. However, despite these kinematic and performance differences, our results showed that rhizostomes and “semaeostomes” travelled the same distance per pulsation cycle throughout development (Fig. 1D). The implications of this finding is that, hydrodynamically, both groups get the same result out of each pulsation cycle. This is true for distance travelled but also probably true for fluid transported to feeding surfaces. However, they go about it very differently. “Semaeostomes” appear to optimize swimming efficiency^8^. As such, they had very long and slow pulsations times (Fig. 1C), and long contraction and relaxation times (Supplementary Fig. S2E,F) which lowered their pulsation frequencies and swimming velocities (Supplementary Fig. S2D, Fig. 1B). Slowing bell kinematics increases efficiency (measured as cost of transport) over rhizostomes^8^. In contrast, rhizostomes appear to favour high fluid processing over swimming efficiency. Having more rapid pulsation times would result in processing more fluid per unit of time. In addition, it would result in higher velocities of the fluid entrained around the bell and transported through capture surfaces^4,20^.

The divergent kinematic strategies between the rhizostomes and the “semaeostomes” are consistent with hydrodynamic requirements of their capture surface morphology and predation strategies. Rhizostomes feed on smaller prey than “semaeostomes”^39–43^ and, therefore, their capture surface morphology is effective for smaller prey^4^. The faster bell kinematics result in faster water velocities around the bell^20^ which facilitate the penetration of fluid through their oral arms and set up the hydrodynamic requirements of sieving and direct interception of small prey by their capture surface^44^. Observations on early development of oral arms suggest that digitata dimensions and spacing are dependent on temperature and fluid regimes^45^, and that such features are regular among different rhizostome species^4^. The similar design features of these structures suggest natural selection for efficient filtration under shared fluid dynamic conditions. In contrast, “semaeostomes” have much lower densities of capture surfaces (i.e. tentacles and oral arms) which enable fluid to circulate through them more freely^17,23,41^. By not having the need to force fluid through a sieve, “semaeostomes” are able to function with slower, more efficient bell kinematics^8^. Strong phylogenetic signal in variables such as Reynolds number and pulsation frequency (Table 2), together with ancestral character analyses (Fig. 4), further corroborate those findings.

Faster relaxation of the bell may be related to the higher collagen content in some rhizostome medusae [e.g*. Rhizostoma pulmo* (Macri, 1778)] that can be up to ten times higher than “semaeostome” medusae (e.g*. Aurelia* spp.)^46^. The mesoglea tissue is composed by collagen fibres that store potential energy from the bell contraction. Since the mesoglea is responsible for bell relaxation and acts antagonistically to the subumbrellar musculature, harder and sturdier bells may generate lower pulsation times, by shortening the relaxation phase. Additionally, pulsation frequencies are independent from body orientation and background flow speed^47^, which indicate that pulsation frequency patterns are probably constrained by physiological and phylogenetic traits, corroborated by the observed high phylogenetic signal (K = 0.89, Table 2).

**Figure 5:**
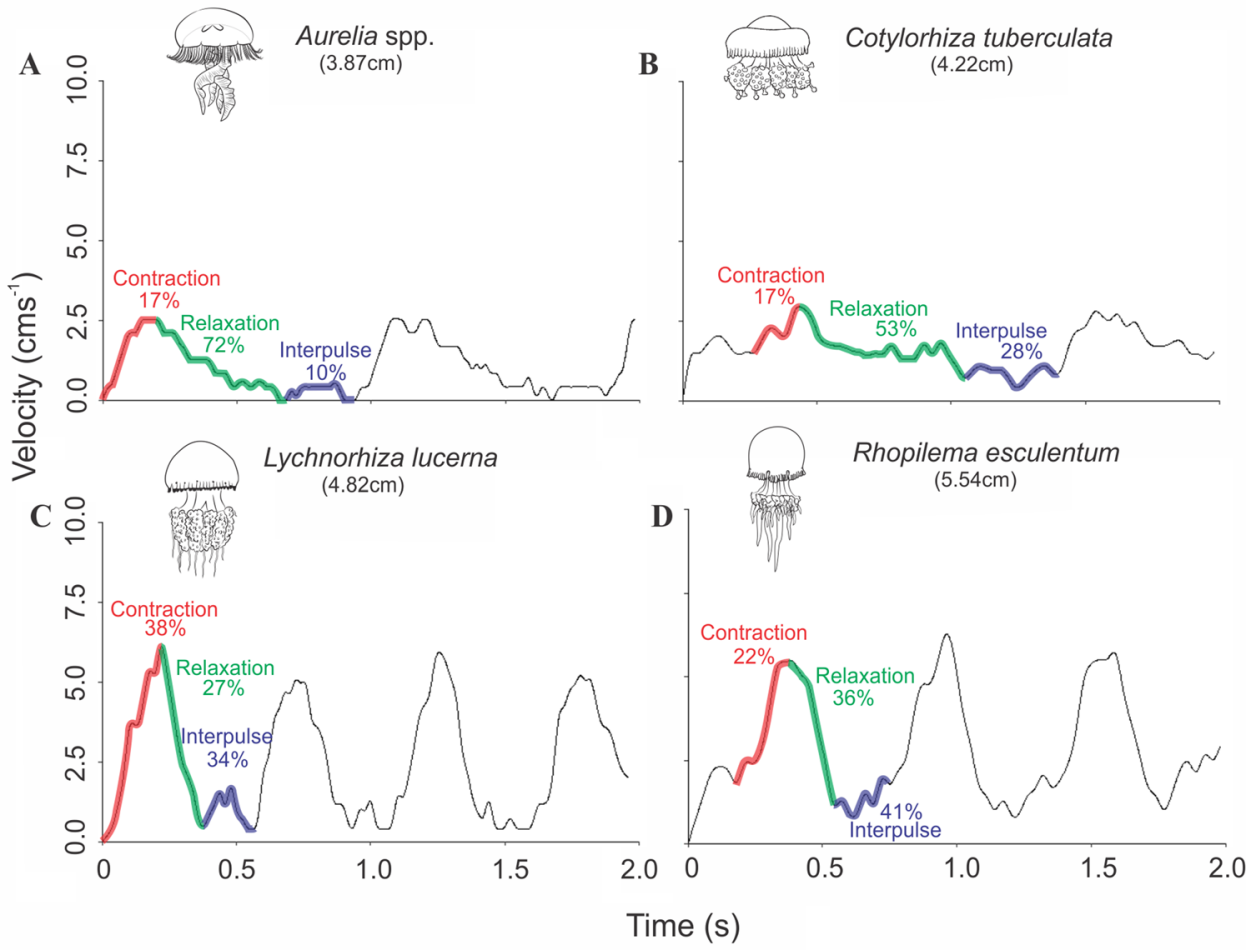
Examples of velocities profiles used in the description of swimming kinematic analysis. (**A**) *Aurelia* spp.; (**B**) *Cotylorhiza tuberculata*; (**C**) *Lychnorhiza lucerna*; (**D**) *Rhopilema esculentum*. Colours indicate each pulsation phase, together with its relative percentage of time spent of the complete pulsation duration, in which: red is the contraction phase, green the relaxation phase and blue the interpulse phase.

From a phylogenetic perspective, our dataset constitutes what is the most comprehensive to date, especially in relation to Discomedusae. Based on our sampling, the phylogenetic analysis recovered Pelagiidae as an early-branching group within Discomedusae and a monophyletic Rhizostomeae resulted as the sister-group of Ulmaridae (Fig. 5)^8^. Additionally, the fact that *L. unguiculata* (Coronatae) medusae had similar patterns with those of Rhizostomeae, could indicate that higher swimming performances are a basal trait in Scyphozoa (but note that this was the only coronate used in our analysis, and that body shape is quite different among the families). Due to low sampling, further investigations should be conducted, especially with the inclusion of more Coronatae taxa, to further elucidate the macroevolutionary dynamics of swimming among scyphozoan lineages. Future species phylogenetic chronogram will enhance our understanding on traits trends. From a temporal point of view, we would give special attention for detected convergence and their historical scenarios: these species would represent proper new models for adaptative conditions on Scyphozoa (i.e. to be combined with developmental biology, ecology and genomics)^48,49^.

Biomimetic experiments with medusae biohybrids (*A. aurita*) manage to alter natural pulsing frequency and swimming velocities, by inserting microelectronics in live animals, with advantages of low power requirements (10–1000 times more efficient than swimming robots)^50^. Such enhanced propulsion (threefold speed increase with twofold metabolic cost) is not naturally exhibited, possibly because its locomotor-feeding system cannot properly function, in addition to being more energetically demanding. Moreover, the increment in speed is compromised in high frequencies because medusae cannot fully relax its bell prior the next contraction^50^. Despite different pulsation frequencies and velocities in scyphomedusae lineages, our data demonstrated similar reached distances per pulsation cycle. We suggest that instead of enhancing medusae pulsation frequency and speed, research should focus in enhancing the use of PER and virtual wall effect, that could even lower power input necessities. Additionally, other scyphomedusae, such as the semeostomids *C. pacifica* and *C. lactea*, and the rhizostomids *C. tuberculata* and *R. esculentum*, that already shows higher use of PER, could be more optimal in biomimetics models.

Ontogeny in scyphomedusae involves remarkable alterations in the bell morphology that lead to different swimming kinematic patterns and performances. While early ephyrae (< 1 cm) had similar patterns, young medusae (> 2 cm) already had clear phylogenetic related swimming, in which rhizostome medusae develop more prolate bells and robust swimming, in terms of velocity and Reynolds numbers, explained by higher pulsation frequencies and shorter pulsation cycles. However, the distance travelled per pulse was virtually the same for rhizostomes and “semaeostomes” despite their observed morphological and kinematic differences. We suggest that the kinematics of the rhizostomes and “semaeostomes” favours different swimming outcomes related to their feeding morphology and strategies. Rhizostome kinematics favours performance to increase flow speeds around their bell to sieve fluid through their dense feeding surfaces. In contrast “semaeostome” kinematics favours efficiency because their less dense feeding structures do not require high velocity flow. The phylogenetic analysis suggests that there are exceptions to these generalities that may reflect different feeding strategies or structure morphologies. Our study presents a framework that broadly compares and establishes some swimming kinematic patterns among scyphomedusae orders with important implications for swimming performances, bioenergetics, feeding behaviour and biomimetics.

## Methodology

### Specimens obtained from cultures and from the field

Most of the specimens were cultivated from polyps that were maintained in darkness, with constant species-specific temperatures, and fed weekly, following similar protocols of Raskoff et al.^51^. Polyps were induced to strobilate by altering specific requirements according with the species, such as culture salinity, temperature, and amount and type of food. Released ephyrae were maintained in pseudokreisels^51^ and fed daily through development for further image sequencing filming.

Some of the specimens of *L. lucerna* and *C. lactea* were collected from the water surface in Cananéia Estuary (~ 24° S) and from Ubatuba beach (~ 23° S), Southeastern Brazil, with hand nets. These specimens were gently transported inside plastic bags to the laboratories of the Center for Marine Biology, in São Sebastião, Brazil, where they were accommodated in 1000 l tanks, feed daily with *Artemia* sp. nauplii and net collected zooplankton, until they were recorded within one or two weeks after samplings.

### Image sequence filming

High-speed images of animals from different geographical distributions around the world were taken with high-speed cameras, such as the Sony NEX FS700 camera, the Photron (FASTCAM SA3) and the KEYENCE VW-9000 high-speed microscope, at rates ranging from 250 to 1000 frames per second. Images were analysed with ImageJ software (National Institute of Health http://imagej.nih.gov/), at time intervals representing minimal body movement or displacement. In addition to the recorded images, published kinematic data^2,4,16,17,19,22,23^ were extracted with the graph digitizer software GetData v2.25.

### Quantification of biomechanics and swimming kinematics

Alterations in bell shape were measured by the instantaneous bell fineness *f*_*i*_:

$${f}_{i}=\frac{{h}_{i}}{{D}_{i}}$$

where *h*_*i*_ (cm) is the instantaneous height of the umbrella and *D*_*i*_ (cm) is the instantaneous bell diameter.

The swimming travelled distance *m* was measured by the changes in positions (*x*, *y*) of the bell apex at intervals times of *t* (s). Instantaneous displacement (*m*_*i*_) was calculated by the Pythagorean theorem:

$${m}_{i}=\sqrt{{({X}_{f}-{X}_{i})}^{2}+{({Y}_{f}-{Y}_{i})}^{2}}$$

where *X*_*f*_ and *Y*_*f*_ and *X*_*i*_ and *Y*_*i*_ are the final and initial positions of the X and Y axes between subsequent images.

Instantaneous velocity (*u*_*i*_) was calculated by:

$${u}_{i}={m}_{i}/{t}_{i}$$

where *m*_*i*_ is the instantaneous displacement (cm) at *t*_*i*_ instantaneous time (s).

Reynolds number describes the fluid regimes around the moving specimens and were calculated by:

$${Re}_{i}=\frac{{D}_{i}{u}_{i}}{v}$$

where *D*_*i*_ is the instantaneous bell diameter (m), *u*_*i*_ is the animal instantaneous velocity (m s^−1^) and *v* the temperature-dependent kinematic viscosity coefficient of salt water, which is temperature dependent (i.e.,* v* = 1.05 × 10^−6^ m^2^ s^−1^ at 20 °C, for instance). For fineness *f*, velocity *u* and Reynolds *Re*, mean and maximum values were extracted, the latter representing the average of maximum values reached per pulse.

For the swimming kinematics analyses, both bell fineness and velocities profiles were used, and the pulsation cycle was split into three phases (Fig. 5): (1) contraction, from the beginning of the pulsation to the highest velocity, that usually coincides with the minimum bell diameter (and highest *f*); (2) relaxation, from the end of the contraction until the expansion leads to the maximum bell diameter (lowest *f*), and usually the lowest velocity; and (3) interpulse, when the medusae presents a velocity gain, before the new contraction cycle, while its bell is still fully expanded (lowest *f*). For each phase of the swimming cycle, mean time (s) and travelled distances (cm) were quantified. Pulsation frequency, or number of pulses per second (Hz), were estimated for sequences with two or more pulses. Pulsation time (s) represented the average time for a complete pulsation cycle. The pulsation distance represented the average distance (cm) reached by each pulsation cycle. The Passive Energy Recapture (PER) phenomenon was quantified by dividing the average travelled distance during the interpulse phase, with the average displacement of the complete pulsation cycle^8,21^.

### Data analyses

All statistical analyses were performed in the open source RStudio software (R version 4.0.3)^52^. To describe how changes in animal’s size was associated with changes in swimming performance, biomechanical and kinematic parameters were used as dependent variables and bell diameter as an independent variable, through linear and non-linear regression analyses. Variables were checked for normality using Shapiro–Wilk test, and log-transformations were performed when necessary. In addition, Bayesian information criteria (BIC) was used to select fitted models with higher parsimony, especially when variable, both logged and non-logged, distributions were not normal (i.e. mean Reynolds number, pulsation frequency, pulsation time, pulsation distance, contraction time, contraction distance, relaxation distance, interpulse time, interpulse distance and passive energy recapture). Regressions were performed separately using orders and families as co-factors and regression lines were estimated together with their respective 95% confidence intervals. Fitted regression models of each variable were gathered in the Supplementary Table S1, along with their respective estimated equations, correlation coefficients (R^2^), p values and degrees of freedom.

To exclude ontogenetic influence, an allometric correction^53^ was performed, by extracting residuals of each species from a regression including all data grouped, for each variable (when p > 0.05). Regressions that best fitted the models were chosen with BIC, and residual mean values for each species were calculated. To describe the relationships among size-normalized biomechanical and kinematic variables, Pearson correlation coefficients (*r*) were calculated (Supplementary Fig. S4), then linear regressions were constructed to variables with significant correlations (p < 0.05). To evaluate which variables had the highest contribution for the differentiation of scyphozoan swimming patterns, a Principal Component Analysis (PCA) was applied, using the *FactoMineR* package^54^.

To identify possible phylogenetic signal, variables were mapped on the Scyphozoa tree and Blomberg’s ‘K’ statistic was estimated^55^ with the phylosig function in R package *phytools*. A strong phylogenetic signal indicates that a trait has likely evolved by gradual changes, such as with a Brownian motion model of evolution. The amount of evolutionary change is proportional to the branch lengths in the tree, thus species with a more recent common ancestor are expected to display more similar traits than more distantly related species. A weak phylogenetic signal suggests that traits are either very stable, or that they are more likely to change. Additionally, the phylogenetic comparative method of ancestral character estimation was performed by contMap function, also in *phytools* package^56^. This analysis estimates ancestral states in each node by techniques of maximum likelihood and interpolates the states along the tree edges^57^.

We estimated a phylogenetic tree from four molecular markers, two nuclear (partial ribosomal 18S and 28S rDNA) and two mitochondrial (partial ribosomal 16S and partial protein-coding cytochrome oxidase I-COI) for 148 validated species of Medusozoa (80 Scyphozoa, 26 Cubozoa, 27 Staurozoa and 15 Hydrozoa) in GenBank (NCBI\nucleotide). Each marker was aligned independently: for the 16S, 18S and 28S markers the MAFFT program E-INS-i strategy was used, and for the COI marker with the MAFFT program codon aware strategy. The alignments were combined in the SequenceMatrix program^58^. Phylogenetic analysis was performed in the IQ-TREE program (Maximum Likelihood criterion, ML), the evolutionary model and optimal partitioning for the considered dataset being initially determined^59,60^. The search for the tree with optimal ML value was performed intensively and four support methods were computed, 2 non-parametric (Bootstrap, SH-aLRT: 1000 pseudo replicates each) and 2 parametric methods (aLRT, aBayes: 1000 replicates each). Because the number of species with data swimming is smaller than those represented in our main phylogeny, a reduced version was considered for the phylogenetic signal study (148 vs 17 terminals). We acknowledge that the group "Semaeostomeae" has not been recovered as monophyletic in our main result and most recent phylogenetic analyses^12,31^; since so far there is no clear hypothesis to replace or update it, we prefer to keep it for the sake of simplicity.

## Supplementary Information

Supplementary Information.

## Data Availability

All data are available in the main text or the supplementary materials.
