# Supplementary material for: Ontogenetic transitions, biomechanical trade-offs and macroevolution of scyphozoan medusae swimming patterns
Source: Sci Rep. 2023 Jun 16;13:9760. doi: 10.1038/s41598-023-34927-w (PMC10276012; doi:10.1038/s41598-023-34927-w)
Supplement: Supplementary file 1 — Supplementary Information. [file 41598_2023_34927_MOESM1_ESM.docx]

**Ontogenetic Transitions, Biomechanical Trade-Offs And Macroevolution Of Scyphozoan Medusae Swimming Patterns**

von Montfort, Guilherme M.*^1^; Costello, John H.^2,9^; Colin, Sean P.^2,3^; Morandini, André C.^4,5^; Migotto, Alvaro E.^5^; Maronna, Maximiliano M.,^4,6^; Reginato, Marcelo^7^; Miyake, Hiroshi^8^; Nagata, Renato M.^1^

^1^Universidade Federal do Rio Grande

^2^Marine Biological Laboratory

^3^Roger Williams University

^4^Universidade de São Paulo

^5^Centro de Biologia Marinha

^6^Universidade Estadual Paulista

^7^Universidade Federal do Rio Grande do Sul

^8^Kitasato University

^9^Providence College

[guibemm@gmail.com](mailto:guibemm@gmail.com),

**Supplementary Material**

**The file includes:**

Figs. S1 to S18

Table S1 and S2


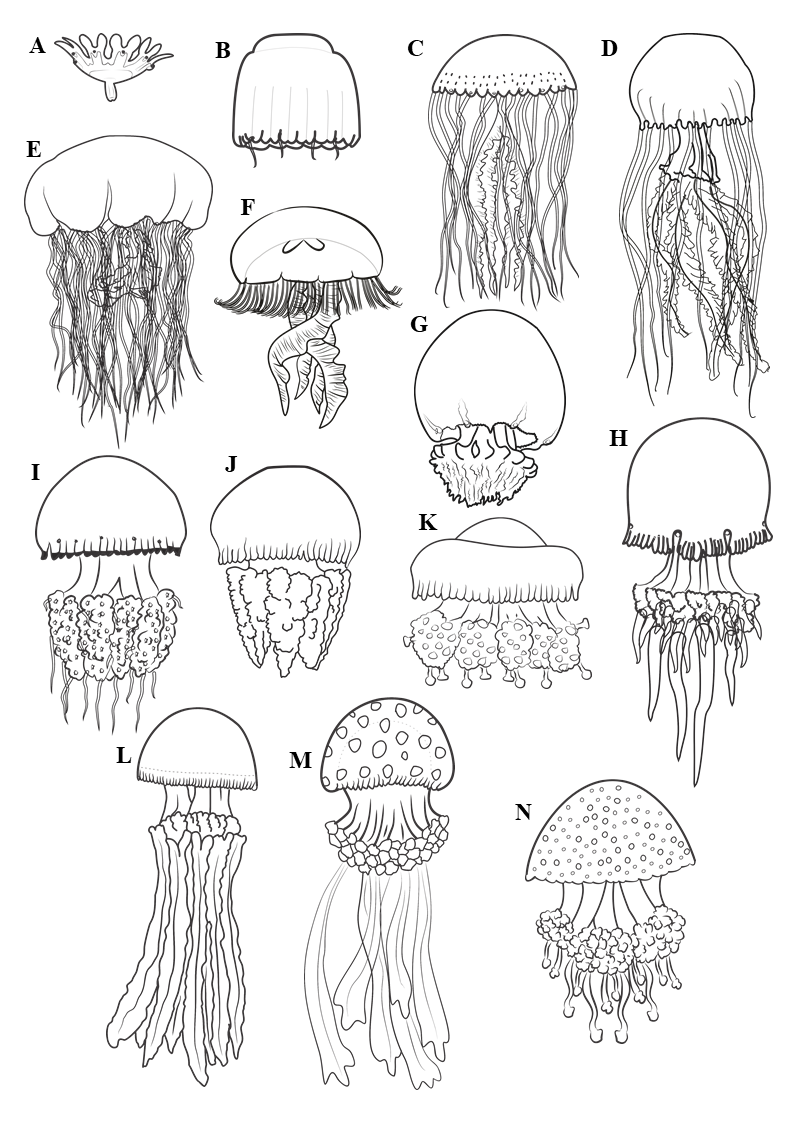


**Supplementary figure S1.** Analysed scyphozoa species and representative morphologies in members of different genera. **A)** Ephyrae generalysed morphology; **B)** Linuche unguiculata; **C)** Sanderia malayensis; **D)** Chrysaora sp.; **E)** Cyanea capillata; **F)** Aurelia spp.; **G)** Stomolophus meleagris; **H)** Rhopilema esculentum; **I)** Lychnorhiza lucerna; **J)** Catostylus mosaicus; **K)** Cotylorhiza tuberculata; **L)** Thysanostoma thysanura; **M)** Mastigias papua; **N)** Phyllorhiza punctata. Based on Jarms et al. (2019) and images of live specimens.


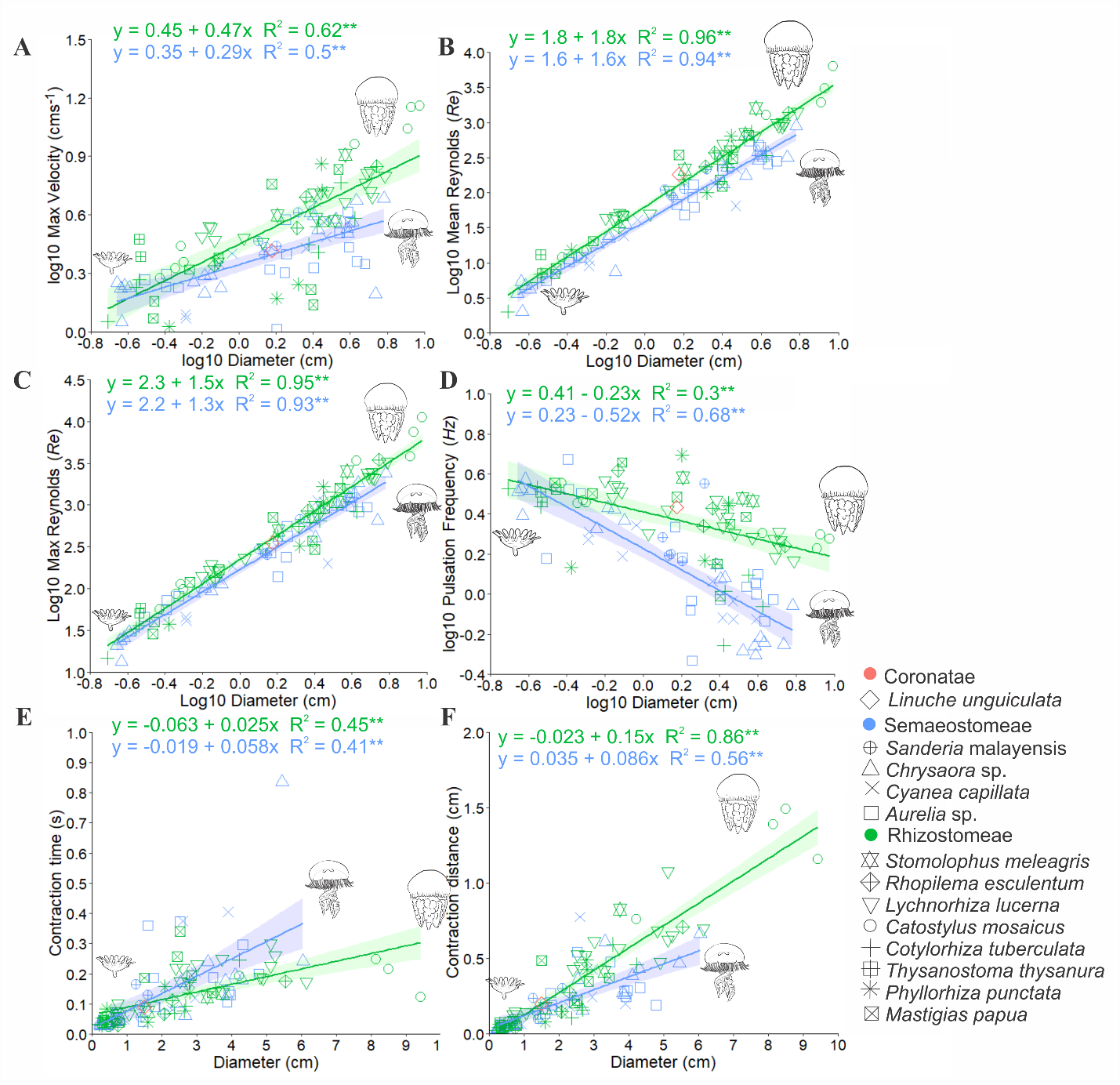


Supplementary figure S2. Scyphozoa orders relationship between diameter (cm) and: A) Maximum velocity; B) Mean Reynolds; C) Maximum Reynolds; D) Pulsation Frequency; E) Contraction time; and E) Contraction distance; Label displays the shape of each plotted species. in red is the Coronatae = *Linuche*; in blue are the Semaeostomeae = *Sanderia*, *Chrysaora*, *Cyanea* and *Aurelia*; in green are the Rhizostomeae = *Stomolophus*, *Rhopilema*, *Lychnorhiza*, *Catostylus*, *Cotylorhiza*, *Thysanostoma*, *Phyllorhiza* and *Mastigias*. Images of representative morphologies were used, common generalized ephyrae, *Aurelia* for Semaeostomeae and *Catostylus* for Rhizostomeae.


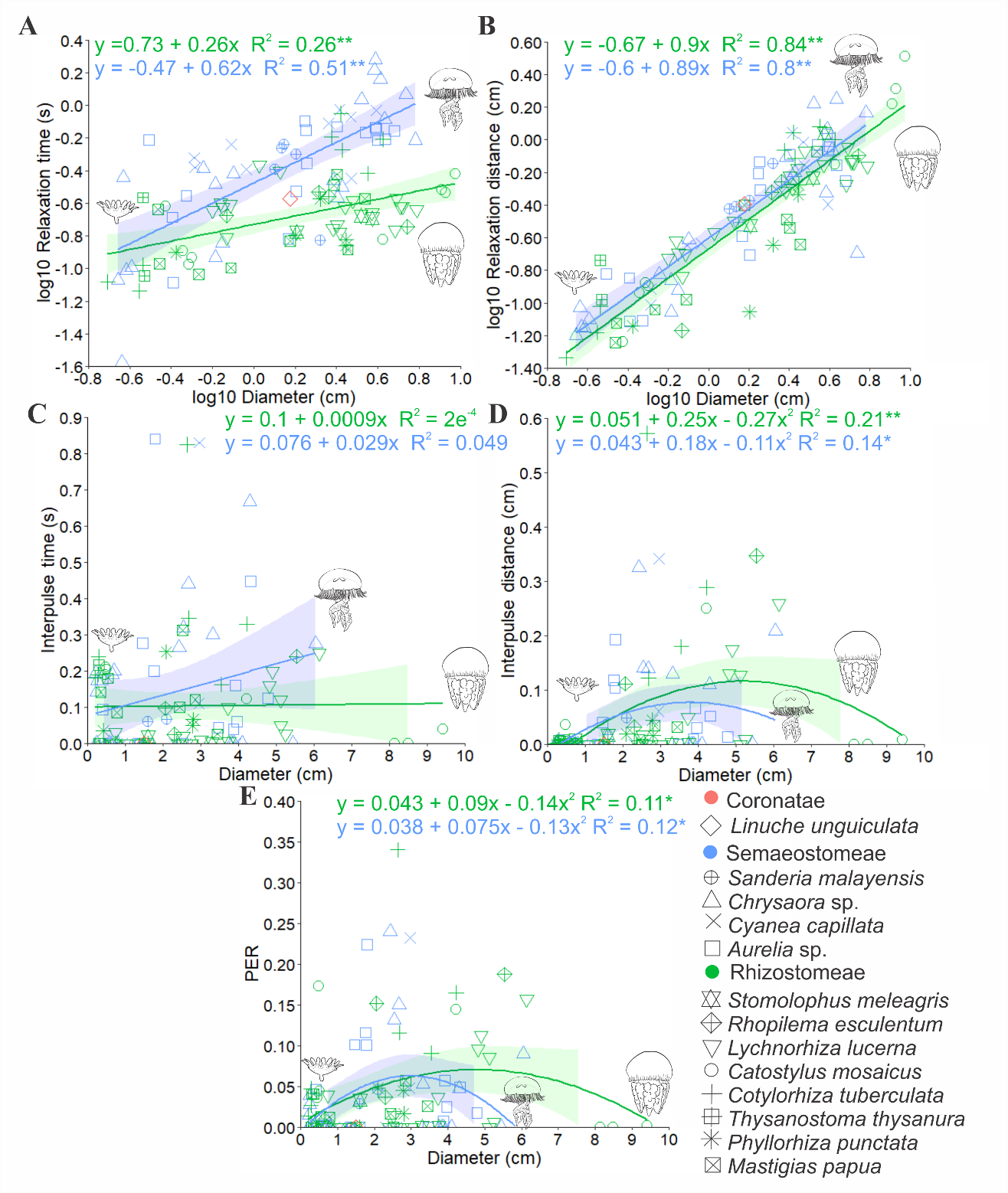


Supplementary figure S3. Scyphozoa orders relationship between diameter (cm) and: A) Relaxation time; B) Relaxation distance; C) Interpulse time; D) Interpulse distance; E) Passive energy recapture; Label displays the shape of each plotted species. in red is the Coronatae = *Linuche*; in blue are the Semaeostomeae = *Sanderia*, *Chrysaora*, *Cyanea* and *Aurelia*; in green are the Rhizostomeae = *Stomolophus*, *Rhopilema*, *Lychnorhiza*, *Catostylus*, *Cotylorhiza*, *Thysanostoma*, *Phyllorhiza* and *Mastigias*. Images of representative morphologies were used, common generalized ephyrae, *Aurelia* for Semaeostomeae and *Catostylus* for Rhizostomeae.


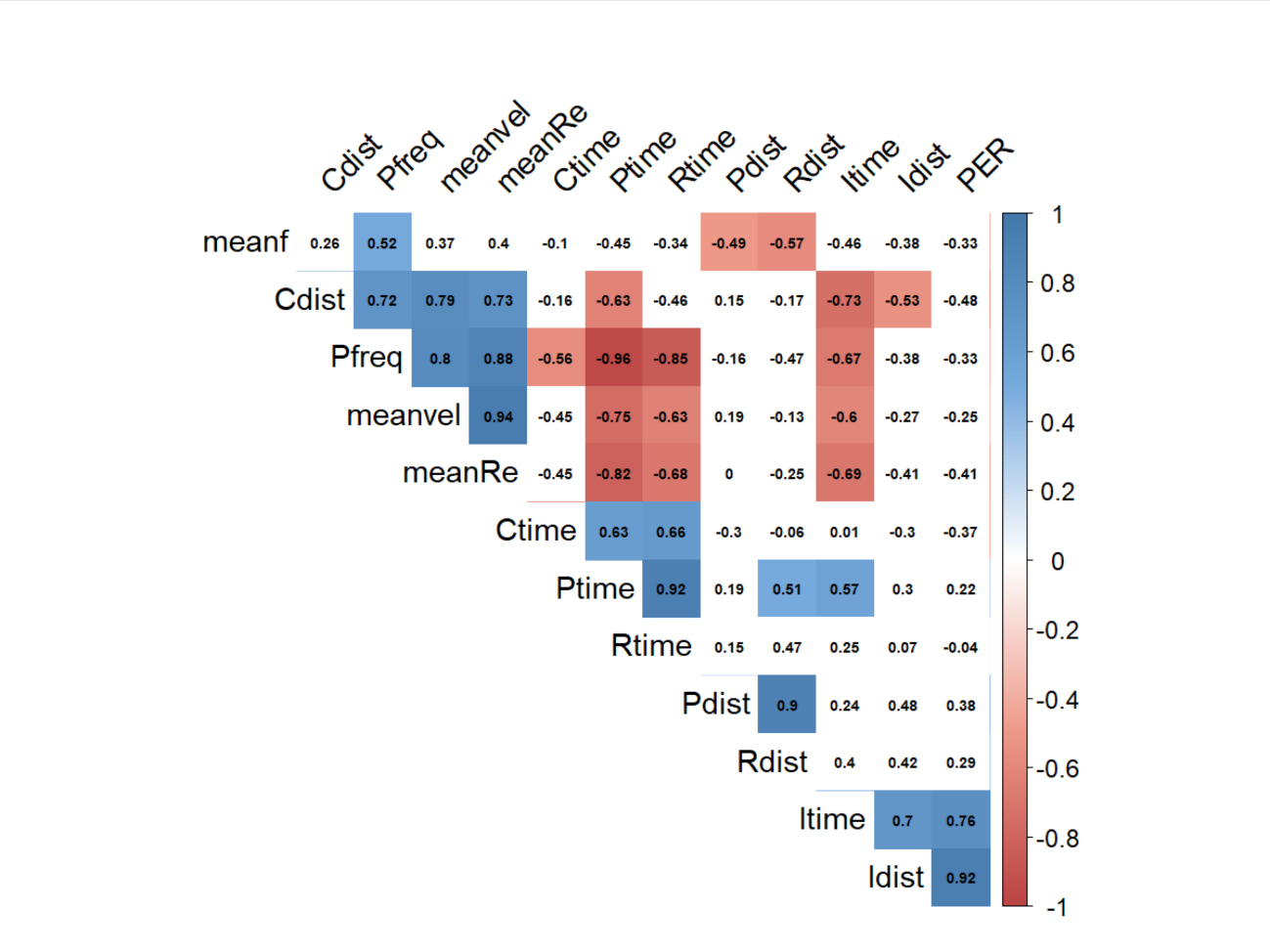


Supplementary figure S4. Biomechanics and kinematics variables of swimming. Scale bar represents Pearson correlation coefficient (*r*), ranging from 1, very high positive correlation, to -1, very high negative correlation. Values inside the squares display coefficient number. Squares in blank represent statistically non-significant coefficient values (p > 0.05).


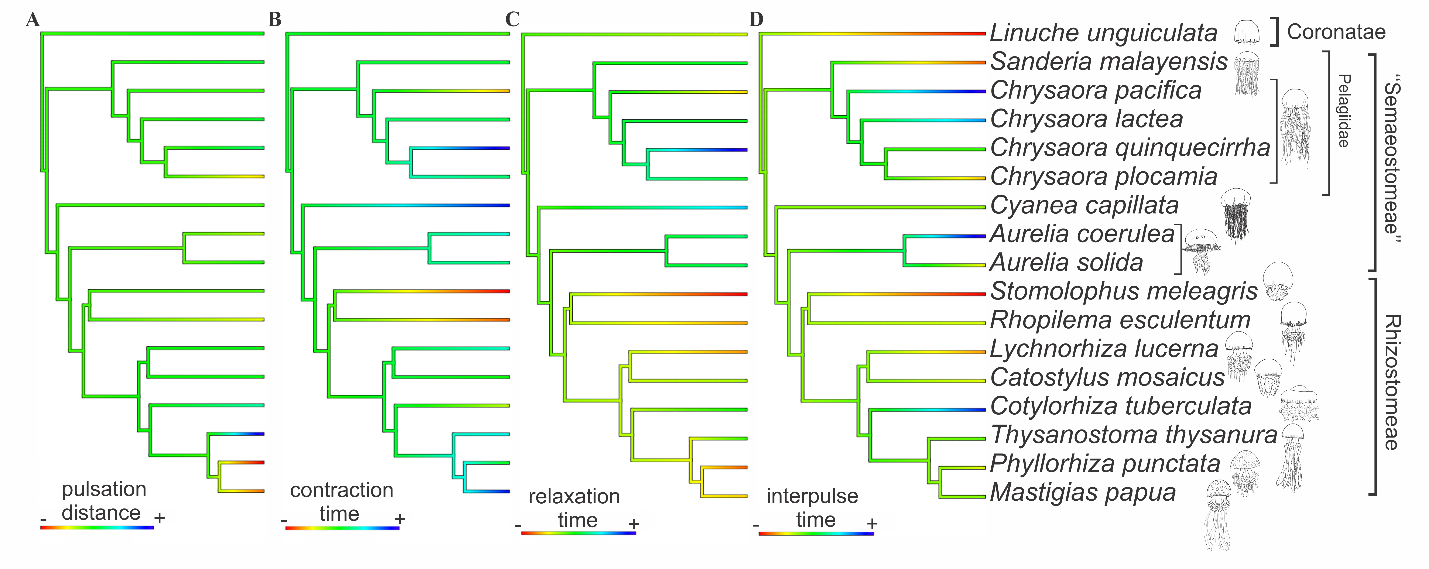


Supplementary figure S5. Ancestral character reconstructions estimated from regression residuals of Scyphozoa: A) pulsation distance; B) contraction time; C) relaxation time; and D) interpulse time. Warm and cool colours on the scale for each variable represent higher and lower values, respectively.


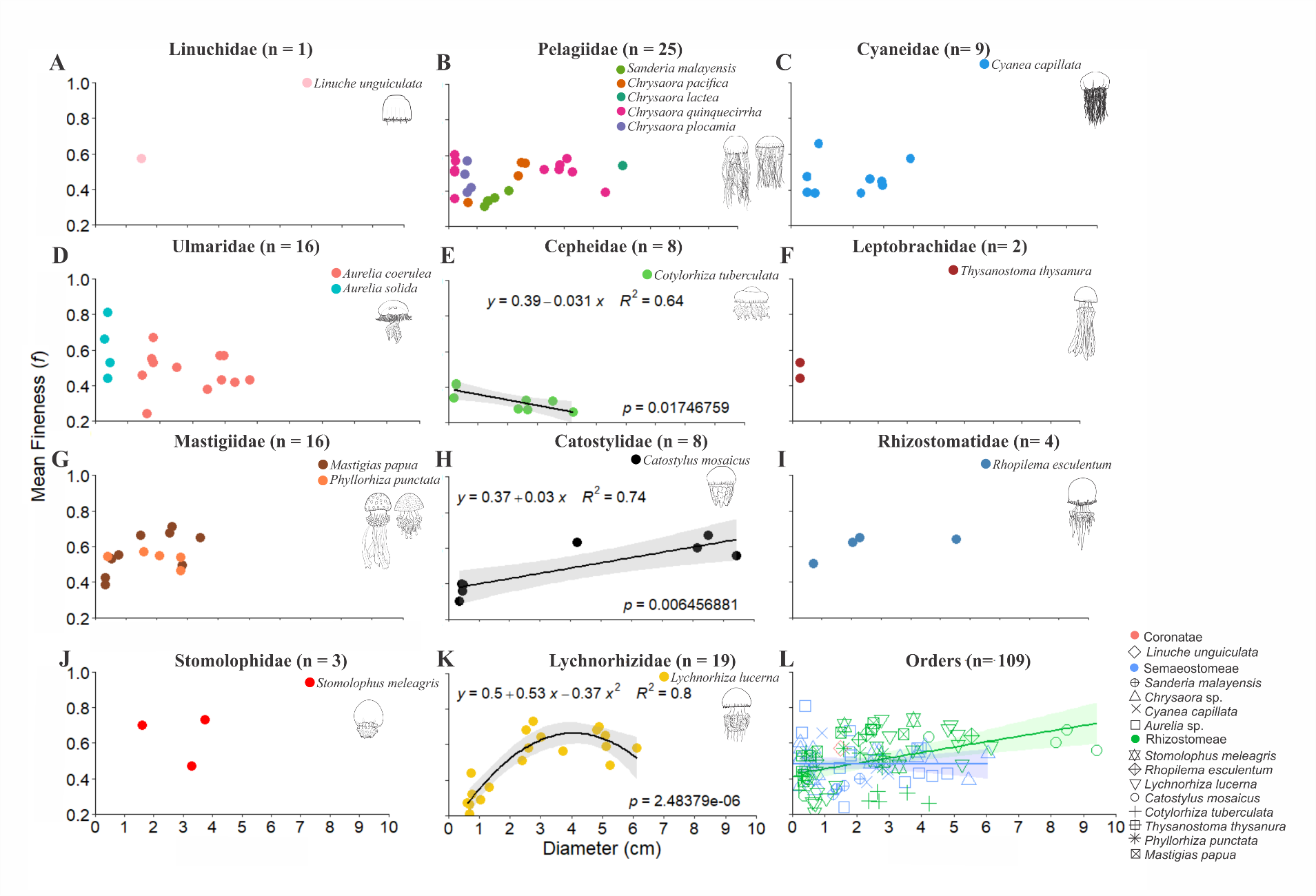


**Supplementary figure S6**. Scyphozoa families’ mean fineness regressions. Regression lines are displayed with confidence intervals of 95%. **A)** Linuchidae; **B)** Ulmaridae; **C)** Pelagiidae; **D)** Cyaneidae; **E)** Cepheidae; **F)** Leptobrachidae; **G)** Mastigiidae; **H)** Catostylidae; **I)** Rhizostomatidae; **J)** Stomolophidae; **K)** Lychnorhizidae; **L)** Orders (red – Coronatae, blue – “Semaeostomeae”, green – Rhizostomeae).


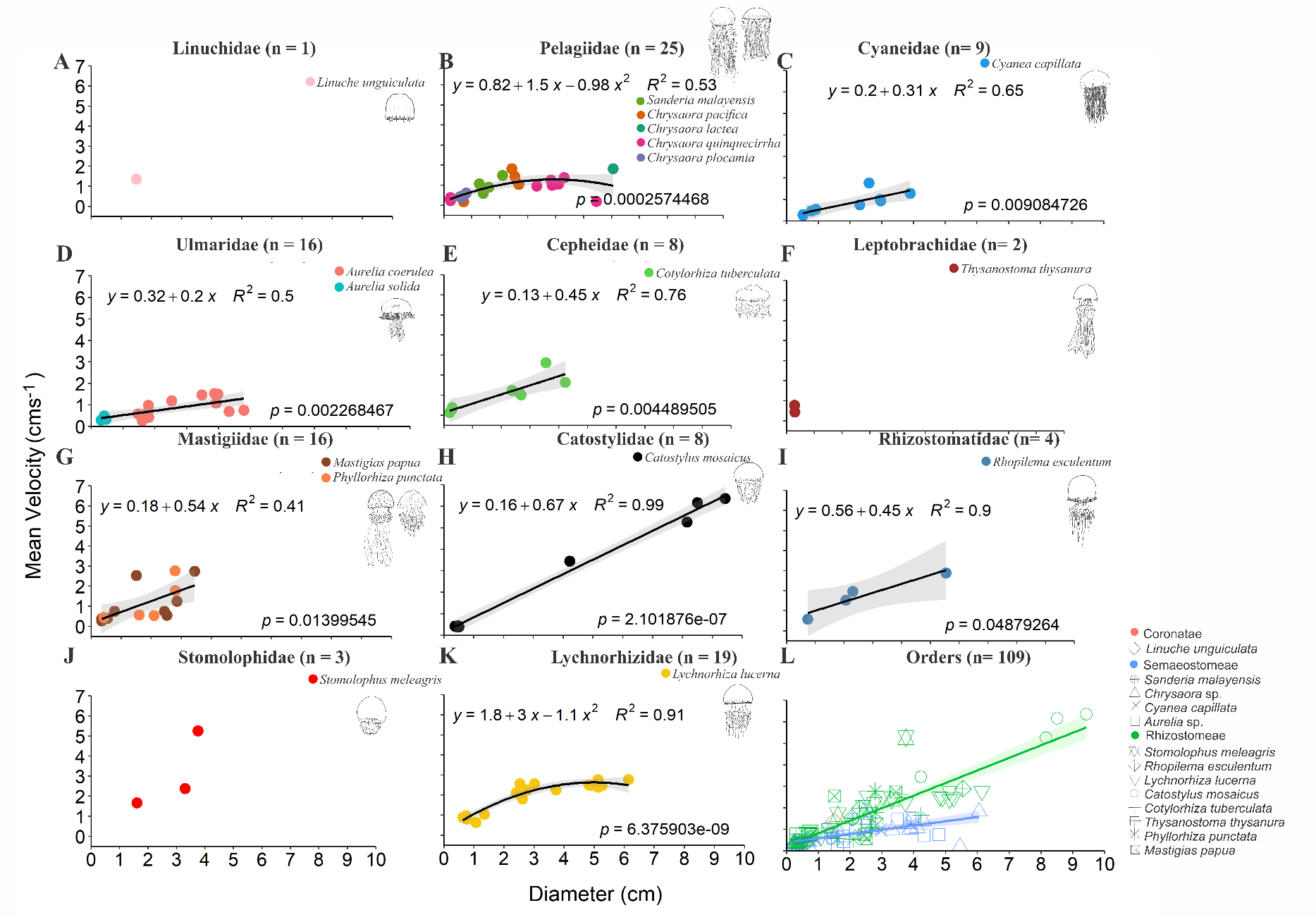


**Supplementary figure S7**. Scyphozoa families’ mean velocity regressions. Regression lines are displayed with confidence intervals of 95%. **A)** Linuchidae; **B)** Ulmaridae; **C)** Pelagiidae; **D)** Cyaneidae; **E)** Cepheidae; **F)** Leptobrachidae; **G)** Mastigiidae; **H)** Catostylidae; **I)** Rhizostomatidae; **J)** Stomolophidae; **K)** Lychnorhizidae; **L)** Orders (red – Coronatae, blue – “Semaeostomeae”, green – Rhizostomeae).


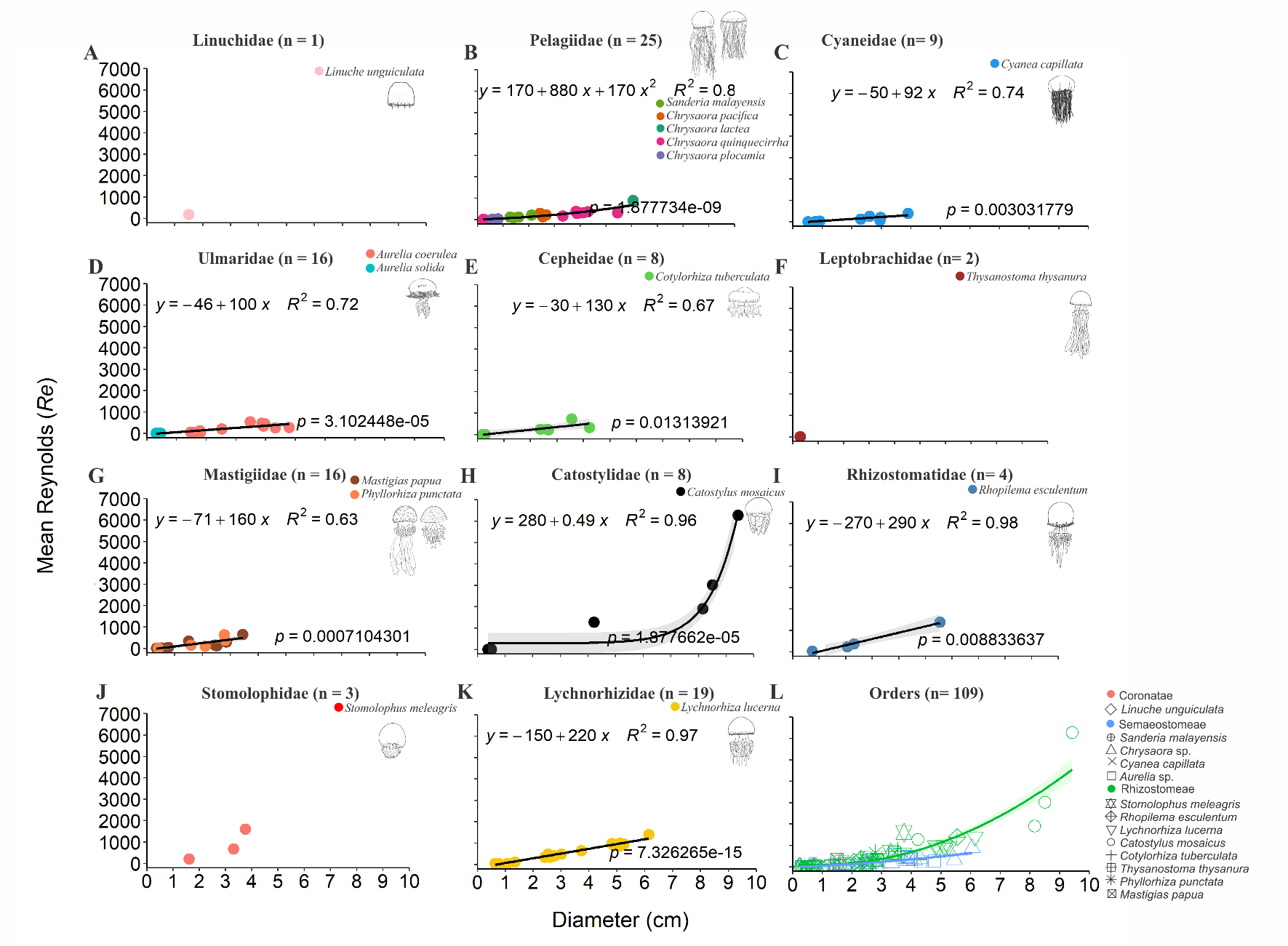


**Supplementary figure S8**. Scyphozoa families’ mean Reynolds regressions. Regression lines are displayed with confidence intervals of 95%. **A)** Linuchidae; **B)** Ulmaridae; **C)** Pelagiidae; **D)** Cyaneidae; **E)** Cepheidae; **F)** Leptobrachidae; **G)** Mastigiidae; **H)** Catostylidae; **I)** Rhizostomatidae; **J)** Stomolophidae; **K)** Lychnorhizidae; **L)** Orders (red – Coronatae, blue – “Semaeostomeae”, green – Rhizostomeae).


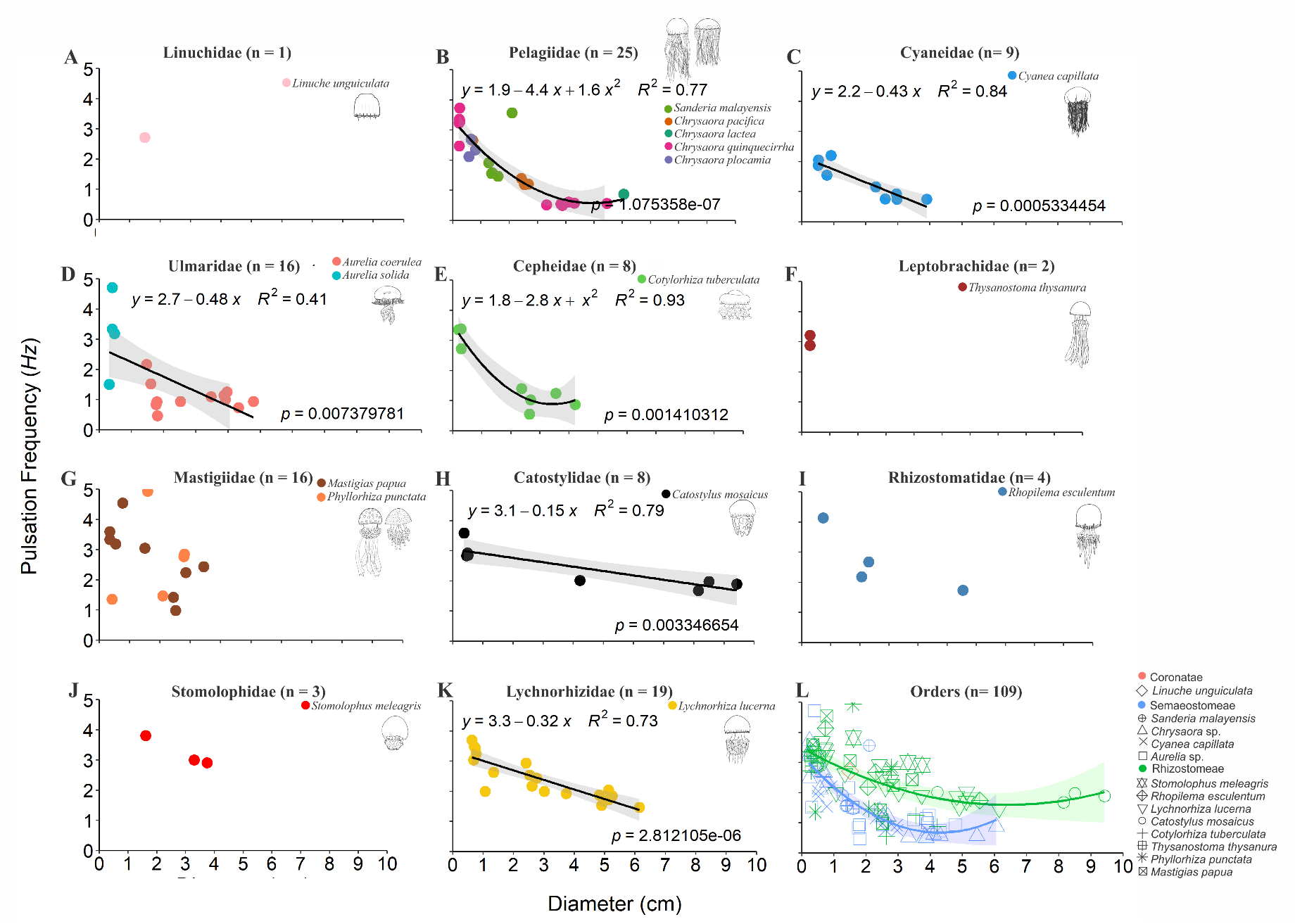


**Supplementary figure S9**. Scyphozoa families’ pulsation frequency regressions. Regression lines are displayed with confidence intervals of 95%. **A)** Linuchidae; **B)** Ulmaridae; **C)** Pelagiidae; **D)** Cyaneidae; **E)** Cepheidae; **F)** Leptobrachidae; **G)** Mastigiidae; **H)** Catostylidae; **I)** Rhizostomatidae; **J)** Stomolophidae; **K)** Lychnorhizidae; **L)** Orders (red – Coronatae, blue – “Semaeostomeae”, green – Rhizostomeae).


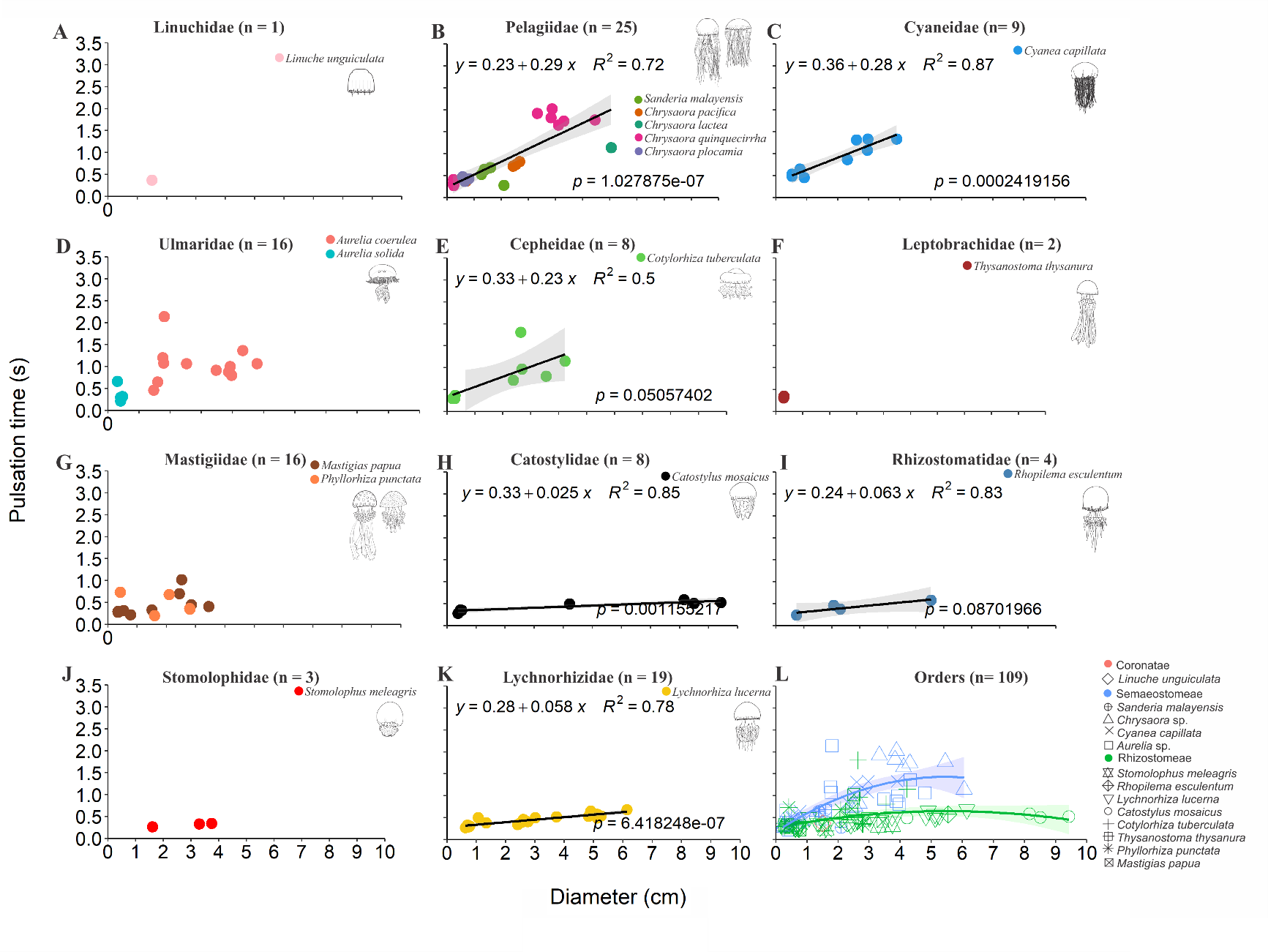


**Supplementary figure S10**. Scyphozoa families’ pulsation time regressions. Regression lines are displayed with confidence intervals of 95%. **A)** Linuchidae; **B)** Ulmaridae; **C)** Pelagiidae; **D)** Cyaneidae; **E)** Cepheidae; **F)** Leptobrachidae; **G)** Mastigiidae; **H)** Catostylidae; **I)** Rhizostomatidae; **J)** Stomolophidae; **K)** Lychnorhizidae; **L)** Orders (red – Coronatae, blue – “Semaeostomeae”, green – Rhizostomeae).


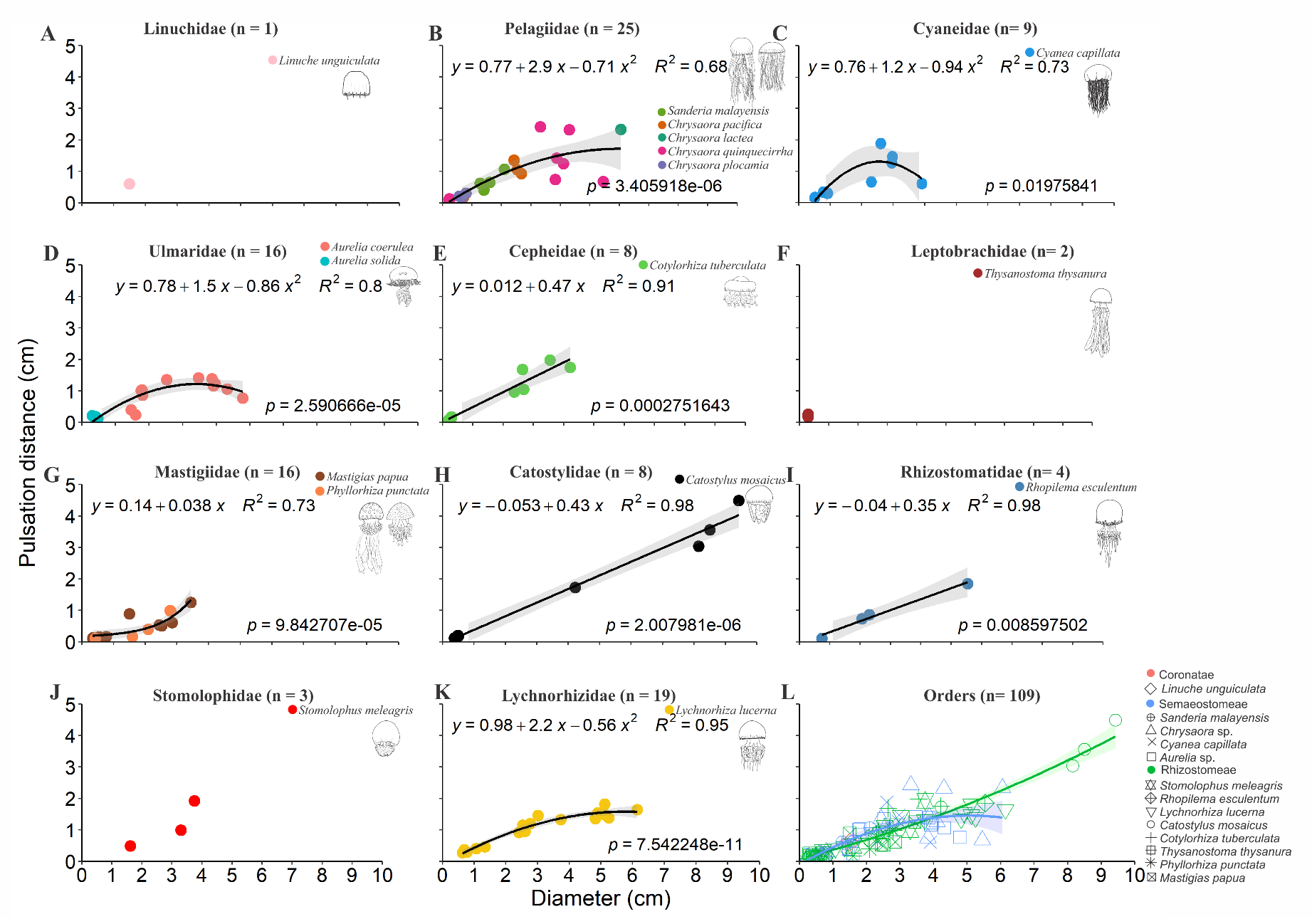


**Supplementary figure S11**. Scyphozoa families’ pulsation distance regressions. Regression lines are displayed with confidence intervals of 95%. **A)** Linuchidae; **B)** Ulmaridae; **C)** Pelagiidae; **D)** Cyaneidae; **E)** Cepheidae; **F)** Leptobrachidae; **G)** Mastigiidae; **H)** Catostylidae; **I)** Rhizostomatidae; **J)** Stomolophidae; **K)** Lychnorhizidae; **L)** Orders (red – Coronatae, blue – “Semaeostomeae”, green – Rhizostomeae).


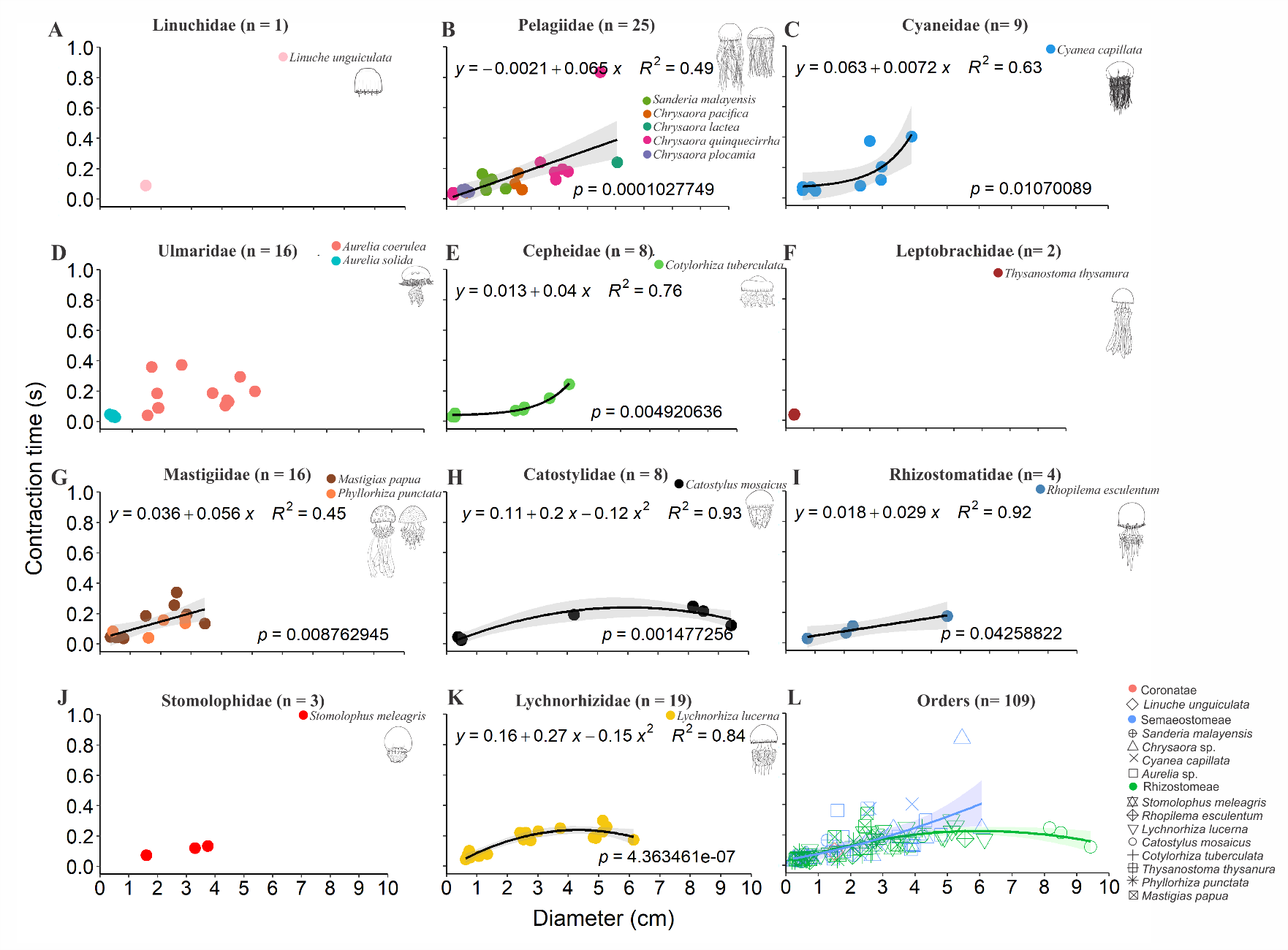


**Supplementary figure S12**. Scyphozoa families’ contraction time regressions. Regression lines are displayed with confidence intervals of 95%. **A)** Linuchidae; **B)** Ulmaridae; **C)** Pelagiidae; **D)** Cyaneidae; **E)** Cepheidae; **F)** Leptobrachidae; **G)** Mastigiidae; **H)** Catostylidae; **I)** Rhizostomatidae; **J)** Stomolophidae; **K)** Lychnorhizidae; **L)** Orders (red – Coronatae, blue – “Semaeostomeae”, green – Rhizostomeae).


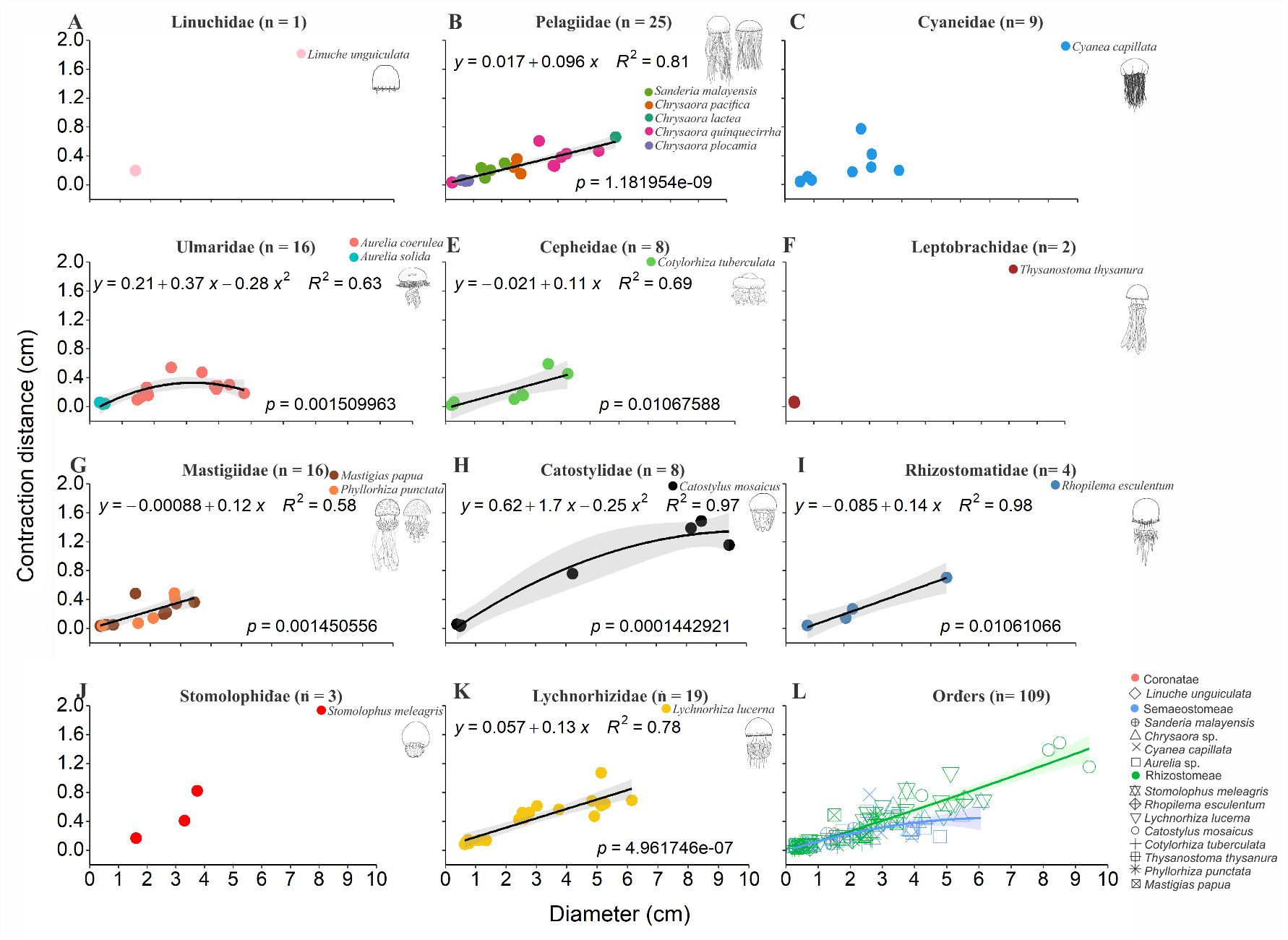


**Supplementary figure S13**. Scyphozoa families’ contraction distance regressions. Regression lines are displayed with confidence intervals of 95%. **A)** Linuchidae; **B)** Ulmaridae; **C)** Pelagiidae; **D)** Cyaneidae; **E)** Cepheidae; **F)** Leptobrachidae; **G)** Mastigiidae; **H)** Catostylidae; **I)** Rhizostomatidae; **J)** Stomolophidae; **K)** Lychnorhizidae; **L)** Orders (red – Coronatae, blue – “Semaeostomeae”, green – Rhizostomeae).


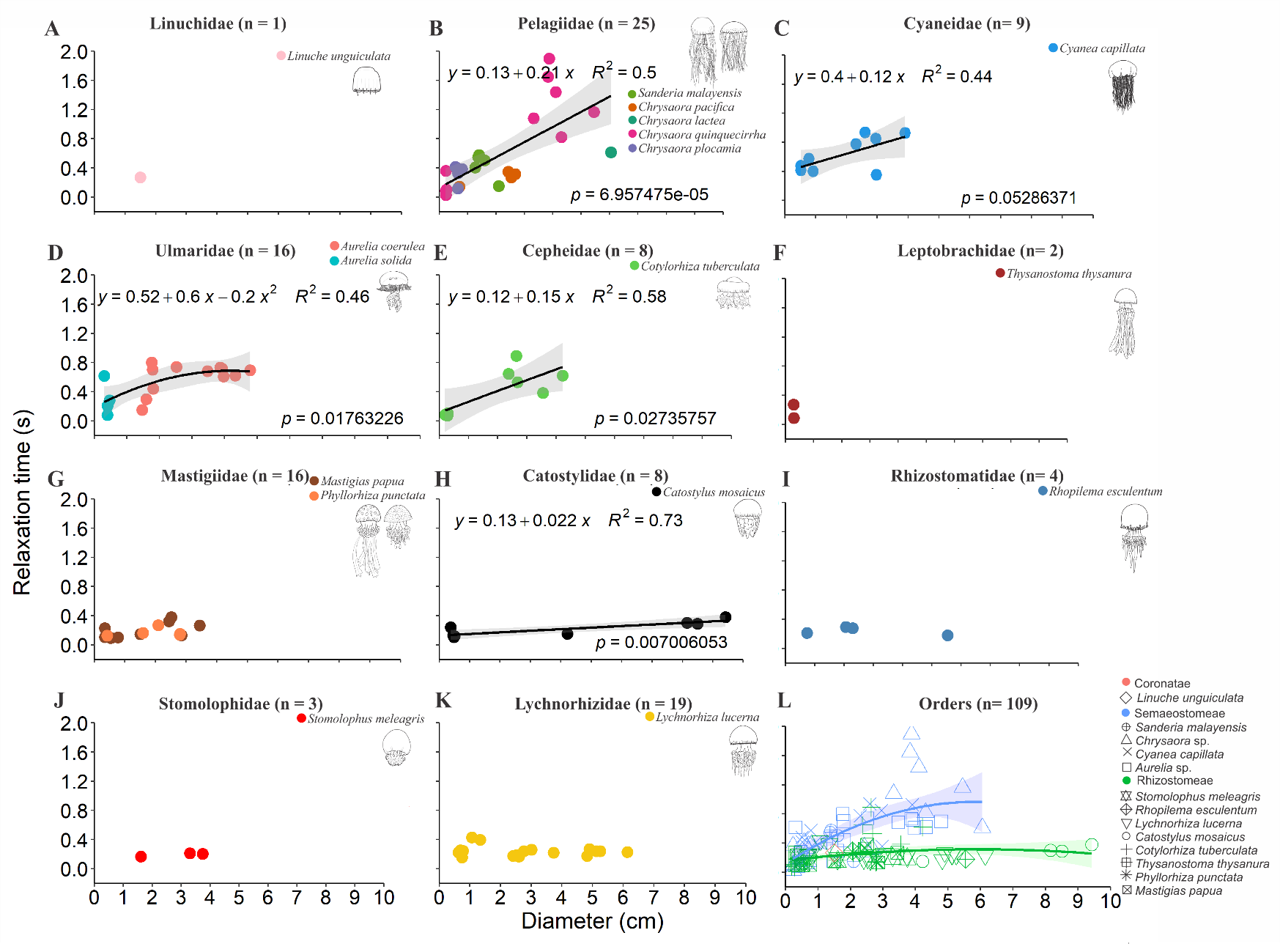


**Supplementary figure S14**. Scyphozoa families’ relaxation time regressions. Regression lines are displayed with confidence intervals of 95%. **A)** Linuchidae; **B)** Ulmaridae; **C)** Pelagiidae; **D)** Cyaneidae; **E)** Cepheidae; **F)** Leptobrachidae; **G)** Mastigiidae; **H)** Catostylidae; **I)** Rhizostomatidae; **J)** Stomolophidae; **K)** Lychnorhizidae; **L)** Orders (red – Coronatae, blue – “Semaeostomeae”, green – Rhizostomeae).


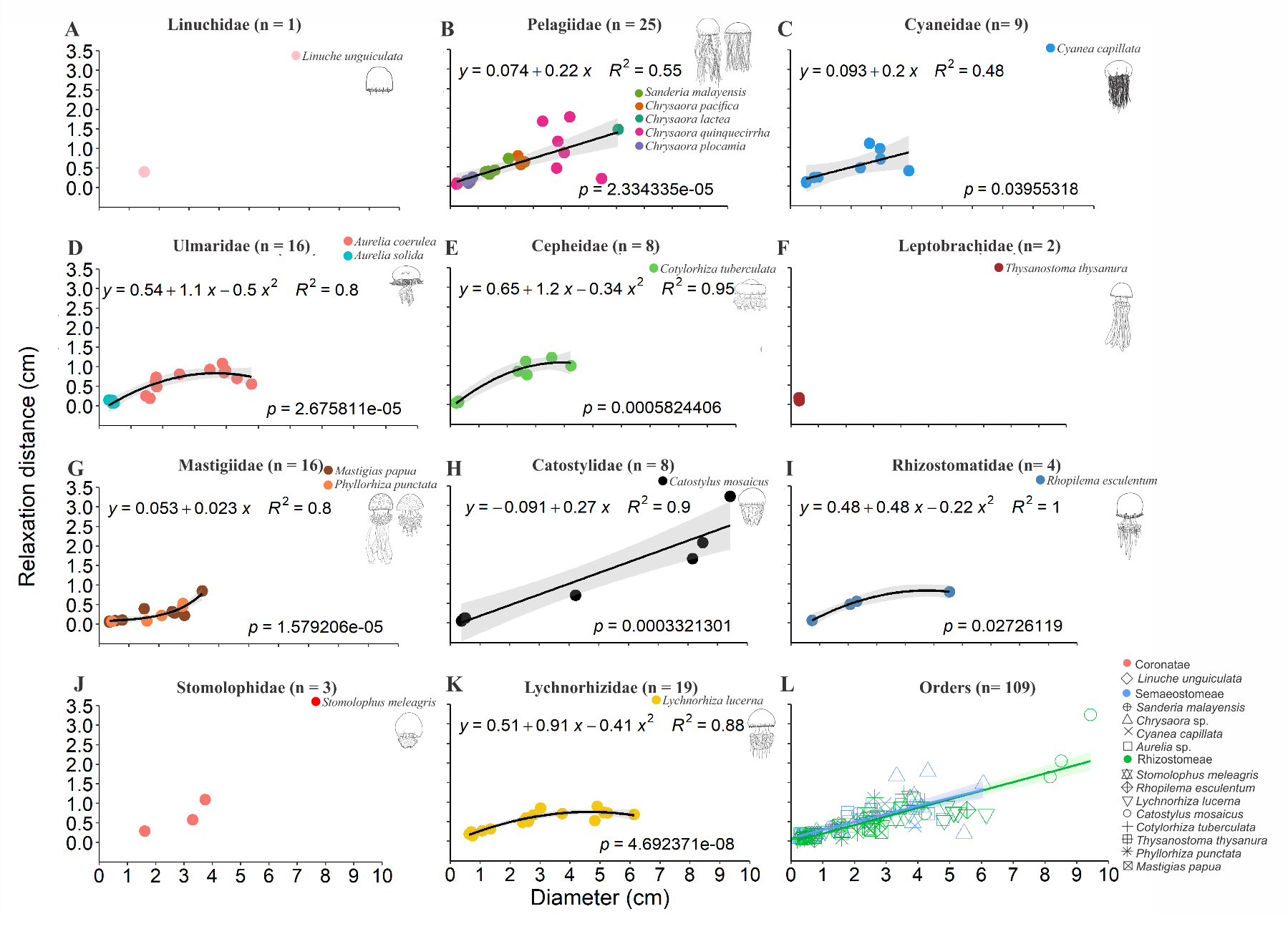


**Supplementary figure S15**. Scyphozoa families’ relaxation distance regressions. Regression lines are displayed with confidence intervals of 95%. **A)** Linuchidae; **B)** Ulmaridae; **C)** Pelagiidae; **D)** Cyaneidae; **E)** Cepheidae; **F)** Leptobrachidae; **G)** Mastigiidae; **H)** Catostylidae; **I)** Rhizostomatidae; **J)** Stomolophidae; **K)** Lychnorhizidae; **L)** Orders (red – Coronatae, blue – “Semaeostomeae”, green – Rhizostomeae).


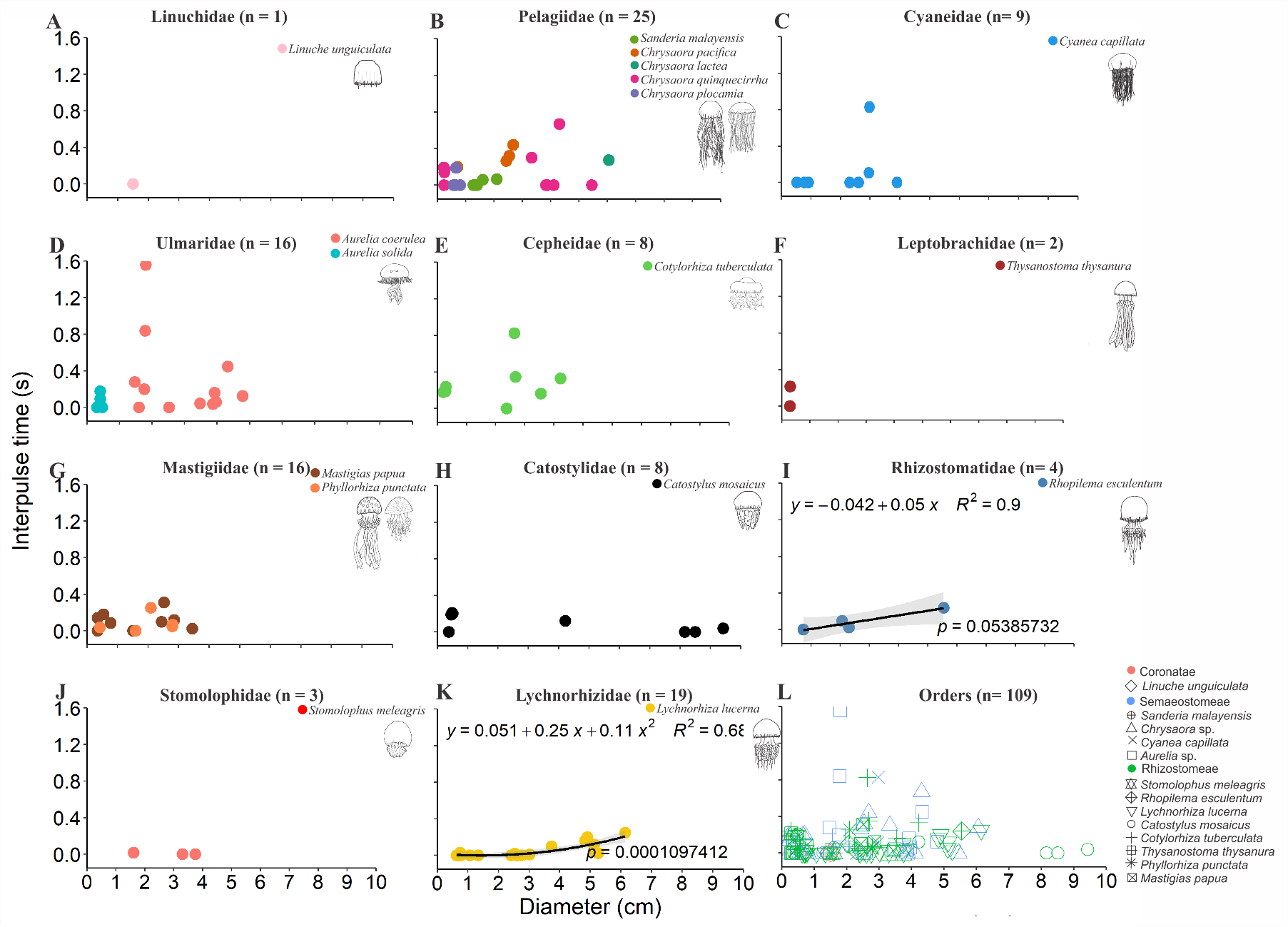


**Supplementary figure S16**. Scyphozoa families’ interpulse time regressions. Regression lines are displayed with confidence intervals of 95%. **A)** Linuchidae; **B)** Ulmaridae; **C)** Pelagiidae; **D)** Cyaneidae; **E)** Cepheidae; **F)** Leptobrachidae; **G)** Mastigiidae; **H)** Catostylidae; **I)** Rhizostomatidae; **J)** Stomolophidae; **K)** Lychnorhizidae; **L)** Orders (red – Coronatae, blue – “Semaeostomeae”, green – Rhizostomeae).


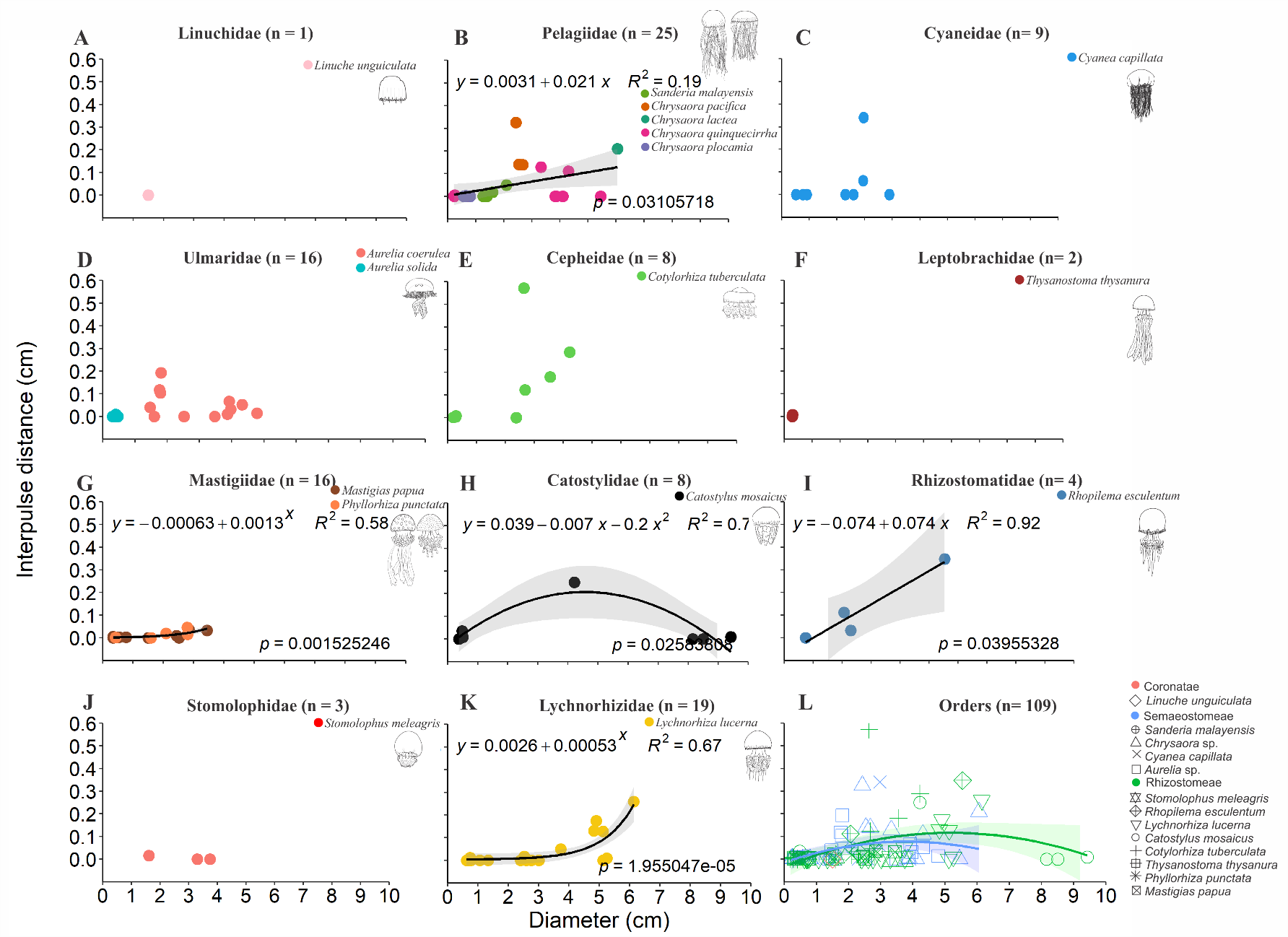


**Supplementary figure S17**. Scyphozoa families’ interpulse distance regressions. Regression lines are displayed with confidence intervals of 95%. **A)** Linuchidae; **B)** Ulmaridae; **C)** Pelagiidae; **D)** Cyaneidae; **E)** Cepheidae; **F)** Leptobrachidae; **G)** Mastigiidae; **H)** Catostylidae; **I)** Rhizostomatidae; **J)** Stomolophidae; **K)** Lychnorhizidae; **L)** Orders (red – Coronatae, blue – “Semaeostomeae”, green – Rhizostomeae).

**
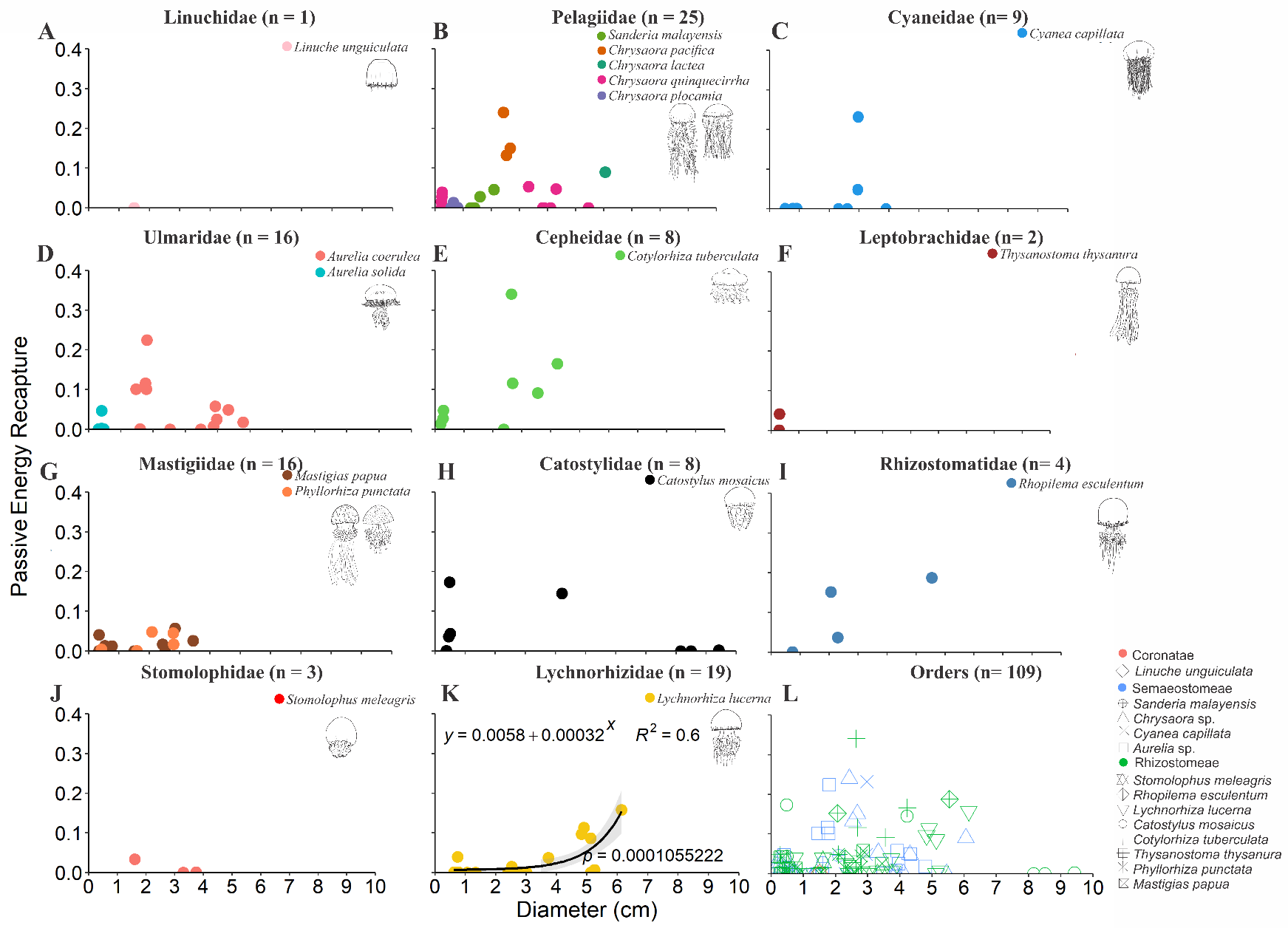
**

**Supplementary figure S18**. Scyphozoa families’ passive energy recapture regressions. Regression lines are displayed with confidence intervals of 95%. **A)** Linuchidae; **B)** Ulmaridae; **C)** Pelagiidae; **D)** Cyaneidae; **E)** Cepheidae; **F)** Leptobrachidae; **G)** Mastigiidae; **H)** Catostylidae; **I)** Rhizostomatidae; **J)** Stomolophidae; **K)** Lychnorhizidae; **L)** Orders (red – Coronatae, blue – “Semaeostomeae”, green – Rhizostomeae).

**Supplementary table S1** – Summary of fitted regressions models along with its estimated equation and correlation coefficient (R^2^). For each model the degrees of freedom were 48 for “Semaeostomeae” and 56 for Rhizostomeae. An asterisk (*) represents that the p value was significant (p < 0.05).

| **Variable (unit)** | **Orders** | **Equation** | **R^2^** |
| --- | --- | --- | --- |
| Mean fineness (f) | "Semaeostomeae" | y = 0.48 + 0.00021x | 0.0000094 |
|  | Rhizostomeae | y = 0.42 + 0.03x | 0.20 * |
| Mean Velocity (cms^-1^) | "Semaeostomeae" | y = -0.25 + 0.48x | 0.79 * |
|  | Rhizostomeae | y = -0.076 + 0.75x | 0.53 * |
| Mean Reynolds (Re) | "Semaeostomeae" | y = 1.6 + 1.6x | 0.94 * |
|  | Rhizostomeae | y = 1.8 + 1.8x | 0.96 * |
| Pulsation Frequency (Hz) | "Semaeostomeae" | y = 0.23 - 0.52x | 0.68 * |
|  | Rhizostomeae | y = 0.41 - 0.23x | 0.30 * |
| Pulsation Time (s) | "Semaeostomeae" | y = -0.23 + 0.52x | 0.68 * |
|  | Rhizostomeae | y = -0.41 + 0.23x | 0.30 * |
| Pulsation Distance (cm) | "Semaeostomeae" | y = -0.43 + 0.91x | 0.84 * |
|  | Rhizostomeae | y = -0.44 + 0.97x | 0.89 * |
| Contraction Time (s) | "Semaeostomeae" | y = -0.019 + 0.058x | 0.41 * |
|  | Rhizostomeae | y = -0.063 + 0.025x | 0.45 * |
| Contraction Distance (cm) | "Semaeostomeae" | y = 0.035 + 0.086x | 0.56 * |
|  | Rhizostomeae | y = -0.023 + 0.15x | 0.86 * |
| Relaxation Time (s) | "Semaeostomeae" | y = -0.47 + 0.62x | 0.51 * |
|  | Rhizostomeae | y = 0.73 + 0.26x | 0.26 * |
| Relaxation Distance (cm) | "Semaeostomeae" | y = -0.6 + 0.89x | 0.80 * |
|  | Rhizostomeae | y = -0.67 + 0.9x | 0.84 * |
| Interpulse Time (s) | "Semaeostomeae" | y = 0.076 + 0.029x | 0.049 |
|  | Rhizostomeae | y = 0.1 + 0.00009x | 0.0002 |
| Interpulse Distance (cm) | "Semaeostomeae" | y = 0.043 + 0.18x - 0.11x^2^ | 0.14 * |
|  | Rhizostomeae | y = 0.051 + 0.25x - 0.27x^2^ | 0.21 * |
| Passive Energy Recapture | "Semaeostomeae" | y = 0.038 + 0.075x - 0.13x^2^ | 0.12 * |
|  | Rhizostomeae | y = 0.043 + 0.09x - 0.14x^2^ | 0.11 * |

**Supplementary table S2.** Principal components analysis variables contributions (Ctr) and correlation (Cor) values for the regressions extracted residuals. The 5 variables that most contributed for each component were highlighted in bold.

|  |  | PC1 | | | PC2 | | | PC3 | | PC4 | |
| --- | --- | --- | --- | --- | --- | --- | --- | --- | --- | --- | --- |
| Variable (unit) | | Abreviations | Ctr | Cor | | Ctr | Cor | Ctr | Cor | Ctr | Cor |
| Mean fineness (*f)* | | meanf | 5,39 | -0,59 | 2,72 | | -0,28 | **6,17** | **-0,34** | **75,32** | **0,66** |
| Mean Velocity (cms^-1^) | | meanvel | **10,47** | **-0,83** | 4,20 | | 0,35 | 3,96 | 0,27 | **3,11** | **0,13** |
| Mean Reynolds (*Re*) | | meanRe | **12,52** | **-0,90** | 1,68 | | 0,22 | 2,02 | 0,19 | 0,11 | 0,03 |
| Pulsation Frequency (*Hz*) | | Pfreq | **14,00** | **-0,96** | 1,64 | | 0,22 | 0,46 | -0,09 | 0,19 | -0,03 |
| Pulsation Time (s) | | Ptime | **12,74** | **0,91** | 2,73 | | -0,28 | 2,53 | 0,22 | **3,05** | **0,13** |
| Pulsation Distance (cm) | | Pdist | 1,12 | 0,27 | **16,93** | | **0,71** | **21,32** | **0,63** | 1,80 | 0,10 |
| Contraction Time (s) | | Ctime | 2,21 | 0,38 | **20,76** | | **-0,78** | 2,85 | 0,23 | 0,20 | -0,03 |
| Contraction Distance (cm) | | Cdist | 9,37 | -0,78 | 0,14 | | 0,06 | **11,67** | **0,47** | 0,98 | -0,08 |
| Relaxation Time (s) | | Rtime | 8,50 | 0,74 | 6,41 | | -0,43 | 8,45 | 0,40 | **7,90** | **0,21** |
| Relaxation Distance (cm) | | Rdist | 4,54 | 0,54 | **7,17** | | **0,46** | **22,25** | **0,65** | **3,23** | **0,14** |
| Interpulse Time (s) | | Itime | **9,76** | **0,80** | 3,70 | | 0,33 | 5,64 | -0,33 | 0,50 | -0,05 |
| Interpulse Distance (cm) | | Idist | 5,18 | 0,58 | **15,76** | | **0,68** | 3,97 | -0,27 | 2,88 | 0,13 |
| Passive Energy Recapture (cm) | | PER | 4,20 | 0,52 | **16,17** | | **0,69** | **8,72** | **-0,40** | 0,72 | 0,06 |
